# Terminal-coupling induced critical eigenspectrum transition in closed non-Hermitian loops

**DOI:** 10.1038/s41598-023-49625-w

**Published:** 2023-12-20

**Authors:** Zhuo Bin Siu, S. M. Rafi-Ul-Islam, Mansoor B. A. Jalil

**Affiliations:** Department of Electrical and Computer Engineering, National University of Singapore, Singapore, 117583 Republic of Singapore

**Keywords:** Phase transitions and critical phenomena, Topological matter

## Abstract

A hallmark feature of non-Hermitian (NH) systems is the non-Hermitian skin effect (NHSE), in which the eigenenergy spectra of the system under open boundary conditions (OBC) and periodic boundary conditions (PBC) differ markedly from each other. In particular, the critical NHSE occurs in systems consisting of multiple non-Hermitian chains coupled in parallel where even an infinitesimally small inter-chain coupling can cause the thermodynamic-limit eigenenergy spectrum of the system to deviate significantly from the OBC spectra of the individual component chains. We overturn the conventional wisdom that multiple chains are required for such critical transitions by showing that such a critical effect can also be induced in a single finite-length non-Hermitian chain where its two ends are connected together by a weak terminal coupling to form a closed loop. An infinitesimally small terminal coupling can induce the thermodynamic-limit energy spectrum of the closed loop to switch from the OBC to the PBC spectrum of the chain. Similar to the critical NHSE, this switch occurs abruptly when the chain length exceeds a critical size limit. We explain analytically the underlying origin of the effect in a Hatano–Nelson chain system, and demonstrate its generality in more complex one-dimensional non-Hermitian chains. Our findings illustrate the generality of critical size-dependent effects in finite NH systems that arise from the interplay between the interfacial boundary conditions and the influence of edge localization.

## Introduction

Non-Hermitian phenomena^1–4^ are one of the most exciting topics that have emerged in condensed matter physics. In general, the breaking of Hermiticity by non- reciprocal couplings between lattice sites or onsite gain/loss terms^5–7^ can induce a plethora of unusual phenomena that include exceptional points^5,8,9^, nodal rings^10^, and the extensive localization of eigenstates^11–15^, also known as the non-Hermitian skin effect (NHSE). The NHSE can be exploited for ultra-sensitive sensors^16,17^, unidirectional transport^18^, and the amplification and attenuation of quantum signals^19,20^, or give rise to interfacial skin modes in non-Hermitian heterojunctions^21^. Furthermore, recently, many exotic non-Hermitian phenomena have been realized in various platforms ranging from optics^22,23^, metamaterials^24,25^, acoustic^26–28^, topolectrical circuits^29–35^ and photonics^36–39^ systems.

In particular, Lee et al. recently uncovered a critical non-Hermitian skin effect (CNHSE) that occurs when two non-Hermitian chains with different NHSE decay lengths are coupled together in parallel, i.e., every site in one chain is coupled to its corresponding site on the other chain^40^. The CNHSE refers to the abrupt transition in the thermodynamic-limit energy spectrum of the system induced by the inter-chain coupling in which even an infinitesimally small inter-chain coupling can cause the thermodynamic-limit energy spectrum to change from the union of the energy generalized Brillouin zones (GBZ) of the individual chains to the GBZ of the combined system, which can deviate substantially from the former. At short lengths and weak inter-chain coupling, the OBC spectrum of such a coupled system resembles the union of the energy GBZs of the two component chains. However, when the length of the chain exceeds a critical interchain-coupling-dependent size, the energy spectrum of the system undergoes a sharp transition and begins to approach the GBZ of the coupled system. We recently analyzed the origin of the CNHSE in a coupled pair of non-Hermitian Hatano–Nelson chains with inverse decay lengths of the same magnitude but opposite signs^11^. In such a system, the OBC eigenenergy spectrum of the coupled system takes the form of a straight line lying strictly on the real energy axis when the system length is below the critical length. When the system length exceeds the critical length, which may be on the order of several tens of lattice sites depending on the interchain coupling strength, the eigenergy spectrum acquires finite imaginary parts and asymptotically approaches the thermodynamic-limit form of an ellipse on the complex energy plane with further increase in the chain length. The existing studies on the CNHSE have so far focused on systems consisting of two non-Hermitian chains with different NHSE decay lengths coupled together^40–42^.

In this work, we show that an analogous critical transition can be induced in even a *single* non-Hermitian chain by connecting the two ends of the chain together with a terminal coupling to form a closed loop. At short lengths of the loop, the energy spectrum of the loop resembles the OBC GBZ spectrum of the open chain. However, even for an infinitesimally small terminal coupling, the energy spectrum of the chain in the thermodynamic limit undergoes a critical transition from the GBZ of the initially open chain to the periodic boundary condition (PBC) spectrum of the initially open chain. Similar to the CNHSE, the switchover of the energy spectrum from the OBC GBZ of the chain to the PBC spectrum occurs sharply when the system length exceeds a critical length. We first illustrate this transition in the most basic example of a non-reciprocal system, i.e., a *single* Hatano–Nelson (HN) chain. We term the transition in the energy spectrum of the system terminal-coupling-induced energy spectrum transition (TCIEST) to distinguish it from the conventional CNHSE arising in multiple coupled chains. We further show the generality of this critical transition in closed non-Hermitian loops, i.e., that the onset of TCIEST is not restricted to a closed HN loop, but also occurs in closed loops of more complicated non-Hermitian systems, indicating that the TCIEST constitutes a general feature of non-Hermitian loops.

## Results

**Figure 1:**
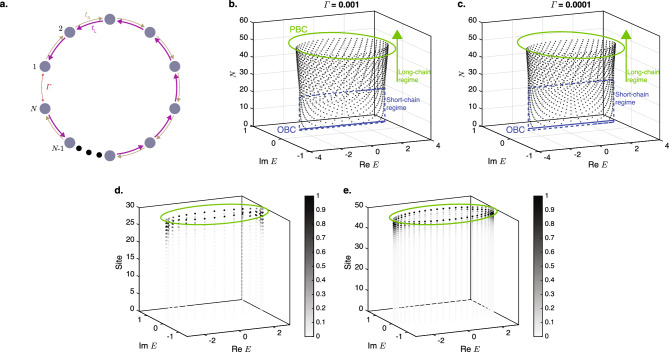
Eigenspectrum and density distribution of closed Hatano–Nelson loops. (**a**) Schematic representation of a closed HN loop containing *N* nodes in which each node from $n=1$ to $n=N-1$ is coupled to its left and right neighbors by the non-reciprocal couplings $t_{\textrm{L}}$ and $t_{\textrm{R}}$, respectively, and the *N*th node is coupled to the first node by the reciprocal terminal coupling $\Gamma$. (**b**,**c**) The eigenenergy spectra of the closed HN loops as functions of the chain length *N* for $t_{\textrm{L}}=2$, $t_{\textrm{R}}=1$ at (**b**) $\Gamma =10^{-3}$, and (**c**) $\Gamma =10^{-4}$. The open-chain OBC and PBC eigenenergy spectra are also respectively shown in blue at the bottom and orange on top for comparison. (**d**,**e**) The eigenenergies and the normalized density distributions across the lattice sites of the eigenstates of (**d**) $N=30$, and (**e**) $N=50$ closed loops with the terminal coupling of $\Gamma =10^{-3}$ together with the PBC eigenenergy spectra. A darker color at each lattice site indicates a larger density at the site.

We consider a closed HN loop (“TC”) where the end points at $n=1$ and $n=N$ of the open HN chain are connected together by a reciprocal terminal coupling $\Gamma$ illustrated in Fig. 1a. The Hamiltonian of a closed HN loop of length *N* is given by

1 $$\begin{aligned} H_{\textrm{TC}} =&\sum ^{N-1}_{j=1} |j\rangle t_{\textrm{R}} \langle j+1| + |j+1\rangle t_{\textrm{L}} \langle j| \nonumber \\&+ \Gamma ( |N\rangle \langle 1| + |1\rangle \langle N|). \end{aligned}$$

The terms in the first row is the Hamiltonian for an open HN chain with the coupling strengths of $t_{\textrm{L}}$ and $t_{\textrm{R}}$ between a site and its left and right neighbors, respectively, and that in the second row the terminal coupling of strength $\Gamma$ between the ends of the chain. $|j\rangle$ ($\langle j|$) is the ket (bra) vector representing a state localized at site *j*.

Figure 1b,c show the eigenvalue spectra of the closed loop in Eq. (1) for the exemplary values of $t_{\textrm{L}}=2$ and $t_{\textrm{R}}=1$ and two different terminal coupling strengths of $\Gamma =10^{-3}$ and $\Gamma =10^{-4}$, respectively. The OBC and PBC spectra of the open chains are also plotted in the figures for comparison. Figure 1b shows that for small values of *N* of less than 22 (henceforth referred to as the short-chain regime), the eigenenergy spectrum takes the form of the open-chain OBC spectrum and is distributed along a line on the real energy axis. When the length of the closed loop exceeds the critical value, its eigenenergy spectrum abruptly deviates from the open-chain OBC spectrum in a manner reminiscent of the CNHSE, and acquires a finite imaginary component. As the length of the closed loop increases, its eigenenergy spectrum approaches that of the open-chain PBC spectrum (henceforth referred to as the long-chain regime), which takes the form of an ellipse on the complex energy plane with the explicit values of $E= ( t_{\textrm{L}}+t_{\textrm{R}})\cos (k) + i ( t_{\textrm{R}}- t_{\textrm{R}})\sin (k)$ for real $\pi < k \le \pi$. Comparing Fig. 1c, which has a smaller value of $\Gamma =10^{-4}$ and critical length of 27, to Fig. 1b with a larger value of $\Gamma =10^{-3}$, it is evident that the transition from the short-chain regime to the long chain regime occurs at longer chain lengths for smaller values of $\Gamma$.

This transition from an OBC-like eigenenergy spectrum to a PBC-like spectrum as the length of the system increases can be understood physically as follows: as the length of the chain increases, the relative proportion of the terminal coupling with the value of $\Gamma$ between sites 1 and *N* compared to the number of non-terminal couplings with the values of $t_{\textrm{L}}$ or $t_{\textrm{R}}$ between the rest of the lattice sites on the chains decreases. The system therefore behaves as if it is homogenously coupled by $t_{\textrm{L}}$ / $t_{\textrm{R}}$ as its length increases, and the deviation of the eigenenergy spectrum and eigenstates of the closed loop from the open-chain PBC spectrum decreases.

At a deeper level, as the length of the closed loop increases, the loop can support modes with increasingly long wavelengths that span over larger numbers of lattice sites. The sensitivity of these long-wavelength modes to the single-site perturbation caused by the deviation of $\Gamma$ from $t_{\textrm{L}}$ and $t_{\textrm{R}}$ decreases with the wavelength of the mode because the length scale of the perturbation is much smaller than its wavelength. Therefore, these long-wavelength modes behave as if the perturbation does not exist, and the corresponding eigenenergy spectrum approaches that of the PBC spectrum. In contrast, in the shorter closed loops, which can only support short-wavelength modes, the relative length scale of the perturbation is proportionally larger for these short-wavelength modes and has a much larger effect on these modes. In particular, because $|\Gamma | \ll |t_{\textrm{L},\textrm{R}}|$, the terminal coupling acts as a break in the connection between the sites, i.e., an OBC, along the chain for the short-wavelength modes. The eigenenergy spectra of the shorter chains therefore resembles that of the open-chain OBC spectra in Fig. 1b,c.

Figure 1d,e show the density distribution at each lattice site (i.e., the square of the absolute value of the wavefunction) for each eigenstate for the chain lengths of $N=30$ and $N=50$, respectively, corresponding to the long-chain regime of Fig. 1b. For both values of *N*, the eigenstates are localized near the right edge of the chain, i.e., at site $n=N$ because of the NHSE. Moreover, comparing Fig. 1d and Fig. 1e, it can be seen that the decay rates of the eigenstates from the right edge towards the interior of the loops are largely similar relative to the respective total lengths of the loops. This is manifestation of the scale-free nature of the TCIEST, which we prove mathematically in the following.

To provide a more rigorous explanation of the above findings, we analyze the NH loop with terminal coupling mathemtically. We begin by recalling some properties of the simplest non-reciprocal 1-dimensional system, i.e., the Hatano–Nelson (HN) chain, to set the stage for subsequent discussion and introduce the notation. The HN chain is a periodically repeating 1-dimensional lattice in which the unit cell consists of only a single site. The non-Hermiticity arises by having the coupling in the left and right directions between two adjacent sites differ from (the complex conjugate of) each other. Thus, the Hamiltonian for a HN chain with *N* nodes numbered 1 to *N* can be written as

2 $$\begin{aligned} H_{\textrm{HN}} = \sum ^{N-1}_{j=1} |j\rangle t_{\textrm{R}} \langle j+1| + |j+1\rangle t_{\textrm{L}} \langle j| \end{aligned}$$

where $|j\rangle$ ($\langle j|$) is the ket (bra) vector representing a state localized at site *j*. For simplicity, we assume that all the coupling constants such as $t_{\textrm{L,R}}$ are real and positive in this paper. The Hamiltonian for the HN chain can also be written in the non-Bloch form as

3 $$\begin{aligned} H_{\textrm{HN}}(\beta ) = t_{\textrm{R}}\beta + t_{\textrm{L}}/\beta \end{aligned}$$

where $\beta \equiv \exp (ik)$ is the non-Bloch factor and *k* is, in general, complex. Under PBC, the values of *k* in $\beta$ are real and range from $-\pi$ to $\pi$. Equation (3) then implies that the loci of the PBC eigenenergies form an ellipse on the complex energy plane with a radius of $t_{\textrm{L}}+t_{\textrm{R}}$ parallel to the real energy axis and a radius of $|t_{\textrm{R}} - t_{\textrm{L}}|$ parallel to the imaginary energy axis. It can be readily obtained from Eq. (3) that for a given energy eigenvalue *E*, the two corresponding values of $\beta$, denoted as $\beta _\pm$, are given by

4 $$\begin{aligned} \beta _\pm = \frac{E \pm \sqrt{E^2 - 4t_{\textrm{L}}t_{\textrm{R}}}}{2 t_{\textrm{R}}}. \end{aligned}$$

In general, the OBC eigenenergy spectrum of a non-Hermitian system is given by the loci of the energy values where the middle two values of $\beta$ (arranged in ascending order of absolute values) have the same absolute values^43^. For the HN chain where there are only two values of $\beta$, this implies that the OBC spectrum is constituted by the values of *E* for which $|\beta _+|=|\beta _-|$. This occurs when *E* is real and the $E^2-4t_{\textrm{L}}t_{\textrm{R}}$ term in the square root of Eq. (4) is negative. The latter implies that the locus of the OBC eigenenergy spectrum is the line $|E|< 2\sqrt{t_{\textrm{L}}t_{\textrm{R}}}, E \in \mathbb {R}$. It also implies that the common value of $|\beta _\pm |=\sqrt{t_{\textrm{L}}/t_{\textrm{R}}}$, and that $E = 2\sqrt{t_{\textrm{L}}t_{\textrm{R}}}\cos (k_+)$ where $k_+\equiv \textrm{arg}(\beta _+)$. The (unnormalized) wavefunction of an OBC eigenstate $\psi _{\textrm{HN}}$ for a finite chain with *N* sites labelled as $n=1,2,\ldots N$ can then be written as

5 $$\begin{aligned} \psi _{\textrm{HN}}(n) = \left( \frac{t_{\textrm{L}}}{t_{\textrm{R}}} \right) ^{\frac{N}{2}} \big (\exp (i n k_+ ) + (-1)\exp (-i n k_+) \big ), \text {where }(N+1)k_+/\pi \in \mathcal {Z} , \end{aligned}$$

from which it can be readily verified that the wavefunction satisfies the boundary conditions of $\psi _{\textrm{HN}}(0)=\psi _{\textrm{HN}}(N+1) = 0$ beyond the boundaries of the chain. It can also be seen from Eq. (5) that the wavefunction is proportional to $(t_{\textrm{L}}/t_{\textrm{R}})^{N/2}$. This implies that when $|t_{\textrm{L}}| > |t_{\textrm{R}}|$, the wavefunction would be exponentially localized near the right edge of the chain, i.e., at $n=N$.

### Short-chain regime

We now consider the closed HN chain (“CHN”) where the end points at $n=1$ and $n=N$ of the open HN chain are connected together by a reciprocal terminal coupling $\Gamma$ illustrated in Fig. 1a . The corresponding Hamiltonian of the closed HN chain is given by

6 $$\begin{aligned} H_{\textrm{CHN}} = H_{\textrm{HN}} + \Gamma ( |N\rangle \langle 1| + |1\rangle \langle N|). \end{aligned}$$

We first explain the breakdown of the short-chain regime at which the loci of the eigenenergies deviate from that of the open-chain OBC energy spectrum and begins to gain finite imaginary parts. Within the interior of the closed chain at $n=2,\ldots , N-1$, each lattice site is coupled both its left and right neighbors by $t_{\textrm{L}}$ and $t_{\textrm{R}}$. Eq. (3) is therefore still applicable to these sites. We find numerically that within the short-chain regime, the wavefunction of an eigenstate at the energy *E* at these interior sites is a generalization of the form adopted by the eigenstates of the open HN loop given in Eq. (5), i.e., it has the generic form of

7 $$\begin{aligned} \psi _{\textrm{CHN}}(n) = \left( \frac{t_{\textrm{L}}}{t_{\textrm{R}}} \right) ^{\frac{n}{2}} \big (\exp (i n k_+ ) + \exp (i\phi )\exp (-i n k_+) \big ),\ n = 2,3,\ldots , N-1 . \end{aligned}$$

In Eq. (7), the original $(-1)$ coefficient of $\exp (-ink_+)$ in Eq. (5) is now generalized into a phase factor $\exp (i\phi )$ with the real-valed phase angle $\phi$ to be determined while the exponential factor of $\left( \frac{t_{\textrm{L}}}{t_{\textrm{R}}} \right) ^{N/2}$ remains unchanged. In contrast to the interior nodes, the nodes at the extremes at $n=1$ and $n=N$ are no longer coupled by $t_{\textrm{L}}$ and $t_{\textrm{R}}$ to both their left and right neighbors because the terminal coupling is replaced by $\Gamma$. We denote the values of the wavefunctions at these two terminal locations in a slightly different notation with $\psi _{\textrm{CHN},1}$ and $\psi _{\textrm{CHN},N}$.

The wavefunctions at the different values of *n* obey the Schrödinger equation

8 $$\begin{aligned} H_{\textrm{CHN}}|\psi _{\textrm{CHN}}\rangle = |\psi _{\textrm{CHN}}\rangle E. \end{aligned}$$

Taking the inner product of Eq. (8) with $\langle n|$ for $n=1,2,N-1$ and *N*, we obtain

9 $$\begin{aligned} \Gamma \psi _{\textrm{CHN}, N} + t_{\textrm{R}}\psi _{\textrm{CHN}}(2)&= E \psi _{\textrm{CHN}, 1}, \end{aligned}$$

10 $$\begin{aligned} t_{\textrm{L}}\psi _{\textrm{CHN}, 1} + t_{\textrm{R}}\psi _{\textrm{CHN}}( 3)&= E \psi _{\textrm{CHN}}(2), \end{aligned}$$

11 $$\begin{aligned} t_{\textrm{L}}\psi _{\textrm{CHN}}(N-2) + t_{\textrm{R}}\psi _{\textrm{CHN},N}&= E \psi _{\textrm{CHN}}(N-1), \end{aligned}$$

12 $$\begin{aligned} t_{\textrm{L}}\psi _{\textrm{CHN}}(N-1) + \Gamma \psi _{\textrm{CHN}, 1}&= E \psi _{\textrm{CHN}, N} . \end{aligned}$$

When $(t_{\textrm{L}}/t_{\textrm{R}})^{N/2} \gg 1$, as is the case here even for all but the smallest values of *N* in the short-chain regime, the $\Gamma \psi _{\textrm{CHN},1}$ term in Eq. (12) is negligible compared to the other two terms (the disparity between the magnitudes of $|\psi _{\textrm{CHN},1}|$ and those of $|\psi _{\textrm{CHN},N}|$ and $|\psi _{\textrm{CHN}}(N-1)|$ can also be seen in Fig. 1d,e) and can be dropped. We therefore obtain $\psi _{\textrm{CHN},N} = (t_{\textrm{L}}/E)\psi _{\textrm{CHN}}(N-1) \propto (t_{\textrm{L}}/t_{\textrm{R}})^{N/2}$. Meanwhile, Eq. (9) can be rearranged into the form of

13 $$\begin{aligned} \Gamma \psi _{\textrm{CHN},N} = E\psi _{\textrm{CHN},1} - t_{\textrm{R}}\psi _{\textrm{CHN}}(2). \end{aligned}$$

Because of its exponential dependence on *N* due to the $(t_{\textrm{L}}/t_{\textrm{R}})^{N/2}$ term, $\psi _{\textrm{CHN},N}$ on the left hand side of Eq. (13) has a much larger absolute value than both the $\psi _{\textrm{CHN},1}$ and $\psi _{\textrm{CHN}}(2)$ terms on the right hand side of the equal sign. The difference between the magnitude of $\psi _{\textrm{CHN},N}$ and those of the $\psi _{\textrm{CHN},1}$ and $\psi _{\textrm{CHN}}(2)$ terms grows with *N*. This implies that there exists an upper limit for *N* beyond which Eq. (13) can no longer be satisfied under the short-chain regime form of the wavefunction in Eq. (7) because of the mismatch between the magnitudes of the terms on the left and right sides of the equation. At the value of *N* where this occurs, the short-chain regime breaks down, and the system transitions into the long-chain regime.

We can be put the above arguments on a firmer mathematical footing by explicitly solving the various terms in Eq. (13). The key idea is that given a value of *N* and some $E = 2 \sqrt{ t_{\textrm{L}}t_{\textrm{R}}}\cos (k_+),\ k_+ \in \mathbb {R}$, we check if the given value of *E* is an energy eigenvalue of the closed HN chain of the given length *N*. To do this, we first substitute Eq. (7) into Eqs. (10) and (11) and solve for $\psi _{\textrm{CHN},1}$ and $\psi _{\textrm{CHN},N}$ in terms of the $\exp (i\phi )$ phase factor in Eq. (7). The expressions for $\psi _{\textrm{CHN},1}$ and $\psi _{\textrm{CHN},N}$ are in turn substituted into Eq. (12) to solve for $\exp (i\phi )$. We thus obtain

14 $$\begin{aligned} \exp (i\phi ) = \frac{ \left( \frac{t_{\textrm{L}}}{t_{\textrm{R}}}\right) ^{N/2}\exp (i 2(N+1)k_+)-\frac{\Gamma }{t_{\textrm{R}}}\exp (i(N+2)k_+)}{-\left( \frac{t_{\textrm{L}}}{t_{\textrm{R}}}\right) ^{N/2} + \frac{\Gamma }{t_{\textrm{R}}}\exp (iNk_+)}. \end{aligned}$$

Because $(t_{\textrm{L}}/t_{\textrm{R}})^{N/2} \gg \Gamma /t_{\textrm{R}}$, the second term in the numerator and denominator are negligible compared to their respective first terms and can be dropped, and we obtain the simplification

15 $$\begin{aligned} \exp (i\phi )\approx -\exp (2i(N+1)k_+) . \end{aligned}$$

Substituting this approximation for $\exp (i\phi )$ into Eq. (13), we obtain

16 $$\begin{aligned} \eta _{\textrm{L}} \equiv \Gamma \psi _{\textrm{CHN},N}&\approx -2i\Gamma \left( \frac{t_{\textrm{L}}}{t_{\textrm{R}}}\right) ^{\frac{N}{2}}\exp (i (N+1))\sin (k_+) \end{aligned}$$

17 $$\begin{aligned} \eta _{\textrm{R}} \equiv E\psi _{\textrm{CHN},1} - t_{\textrm{R}}\psi _{\textrm{CHN}}(2)&\approx t_{\textrm{L}}(1-\exp (2i(N+1)k_+)) \end{aligned}$$

for the left and right sides of the equation, respectively.

**Figure 2:**
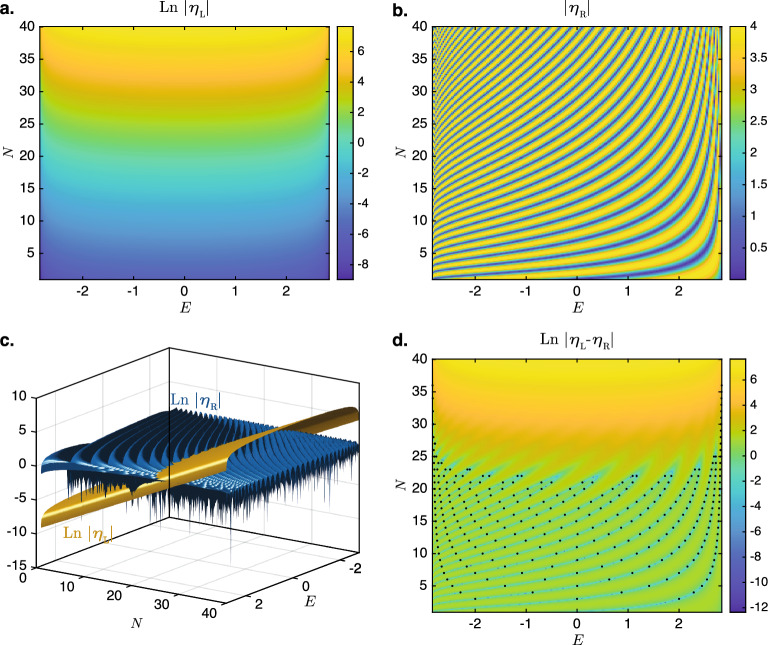
(**a**) $\textrm{Ln}\ |\eta _{\textrm{L}}|$ and (**b**) $|\eta _{\textrm{R}}|$ plotted as functions of *N* and *E*. Note that the *logarithm* of $|\eta _{\textrm{L}}|$ is plotted in (**a**) while the value of $|\eta _{\textrm{R}}|$ itself is plotted in (**b**). (**c**) The surface plots of $\textrm{Ln}\ |\eta _{\textrm{R}}|$ and $\textrm{Ln}\ |\eta _{\textrm{L}}|$ plotted together to show the values of *N* at which $\textrm{Ln}\ |\eta _{\textrm{R}}|$ and $\textrm{Ln}\ |\eta _{\textrm{L}}|$ can possibly intercept each other. (**d**) $\textrm{Ln}|\eta _{\textrm{L}}-\eta _{\textrm{R}}|$, which approaches negative infinity when $\eta _{\textrm{L}} = \eta _{\textrm{R}}$. The eigenvalues of $H_{\textrm{CHN}}$ that fall on the real axis are also shown as black dots at integer values of *N* in this plot. Parameters used: $t_{\textrm{L}}=2$, $t_{\textrm{R}}=1$, and $\Gamma =10^{-3}$.

The validity of these expressions for $\eta _{\textrm{L}}$ and $\eta _{\textrm{R}}$ are numerically verified in Fig. 2, in which the absolute values of $\eta _{\textrm{L}}$ and $\eta _{\textrm{R}}$ are computed using the exact expression for $\exp (i\phi )$ in Eq. (14) and shown in panels a and b of the figure, respectively. (For the calculations in Fig. 2, *N* is treated as a continuous parameter rather than a discrete integer variable.) Figure 2a shows that $\eta _{\textrm{L}}$ grows exponentially with *N* and varies in a non-oscillatory manner with *E* in agreement with Eq. (16). On the other hand, Fig. 2b shows the oscillatory behavior of $|\eta _{\textrm{R}}|$ oscillates between 0 and $2t_{\textrm{L}}$ with the variation of *E* (recall that *E* and $k_+$ are related through $E = 2\sqrt{t_{\textrm{L}}t_{\textrm{R}}}\cos (k_+)$).

For the given *E* to be an eigenenergy of the closed NH loop of length *N*, $\eta _{\textrm{L}}$ and $\eta _{\textrm{R}}$ must match. However, except at $|E|=2\sqrt{t_{\textrm{L}}t_{\textrm{R}}}$ where the $\sin (k_+)$ term in Eq. (16) vanishes, $\eta _{\textrm{L}}$ grows exponentially with *N*. In comparison, the maximum possible magnitude of $\eta _{\textrm{R}}$ is $2t_{\textrm{L}}$. There is therefore an upper limit for *N* beyond which the values of $\eta _{\textrm{L}}$ and $\eta _{\textrm{R}}$ can no longer match. This match is shown more clearly in Fig. 2c where the magnitudes of the $\eta _{\textrm{L}}$ and $\eta _{\textrm{R}}$ are plotted together on the same scale. As can be seen for large values of *N* (above 25 say), the values of $|\eta _{\textrm{L}}|$ far exceed those of $|\eta _{\textrm{R}}|$ and there is no overlap between them. This signifies the breakdown of the short-chain regime. A clearer depiction of the matching between $\eta _{\textrm{L}}$ and $\eta _{\textrm{R}}$ over the various values of *N* and *E* is given in Fig. 2d, which plots the absolute value of the difference between the two quantities. The blue curves depict regions of very low values of $\textrm{Ln}|\eta _{\textrm{L}}-\eta _{\textrm{R}}|$ at which the two quantities match very closely, while the dots represent the eigenvalues of the closed chain that have real values at integer values of *N*. Because of the proportionality of $\eta _L$ to $\sin (k_+) = \sqrt{1 - E^2/(4t_{\textrm{L}}t_{\textrm{R}})}$, the transition to the long chain regime occurs at slightly larger values of *N* for larger values of |*E*| in Fig. 2d. Incidentally, the proportionality of $\eta _{\textrm{L}}$ to $\Gamma$ implies that the value of *N* at which the transition occurs would be proportional to $\textrm{Ln}(1/|\Gamma |)$. In other words, for smaller values of the terminal coupling $\Gamma$, the critical transition occurs for larger chain length *N*. This trend can also be seen by comparing Figs. 1b and 1c. We will now proceed to study the behavior of the closed HN chains in the long-chain regime beyond the critical transition.

### Long-chain regime

We saw in the previous section that the transition to the long-chain regime occurs when a wavefunction of the form Eq. (7), which is applicable only for real $|E| < \sqrt{4t_{\textrm{L}}t_{\textrm{R}}}$, can no longer satisfy the boundary condition in Eq. (13). When this occurs, the eigenenergy *E* gains an imaginary component, and the two solutions for $\beta$ in Eq. (4) at the eigenenergy *E* would no longer have the same absolute values. In the remainder of this section, we denote the $\beta$ value where $|\beta |$ is closer to 1 as $\beta _1$, and the other value of $\beta$ as $\beta _2$. For any arbitrary value of *E* , Eq. (4) implies that $\beta _1$ and $\beta _2$ are related by

18 $$\begin{aligned} \beta _2 = \frac{t_{\textrm{L}}}{t_{\textrm{R}}}\frac{1}{\beta _1}, \end{aligned}$$

and have complex polar angles of opposite signs. Consequently, introducing $k_1 = \textrm{arg}(\beta _1)$, following Eq. (7), the wavefunction in the interior of the closed chain at $n=2,\ldots N-1$ can be written as

19 $$\begin{aligned} \psi _{\textrm{CHN}}(n) = b_1 |\beta _1|^n \exp (i n k_1) + b_2 |\beta _2|^n \exp (-i n k_1),\ n=2,\ldots ,N-1. \end{aligned}$$

where $b_1$ and $b_2$ are the corresponding weightages of the states associated with $\beta _1$ and $\beta _2$. Without losss of generality, we set $b_1=1$ in the subsequent discussion and let $b_2$ be an as yet undetermined variable to be solved for, and retain the notations of $\psi _{\textrm{CHN},1}$ and $\psi _{\textrm{CHN}, N}$ for the wavefunctions at sites 1 and *N*, respectively.

**Figure 3:**
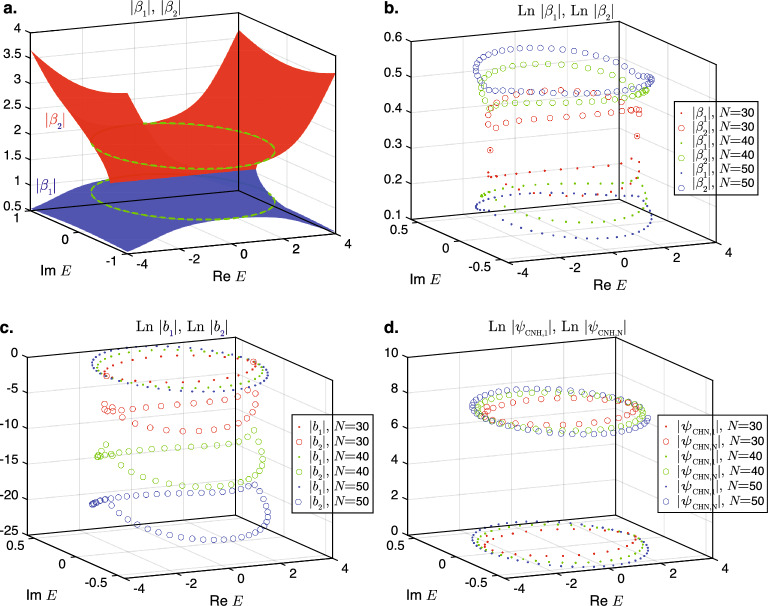
(**a**) The absolute values of $|\beta _1|$ and $|\beta _2|$ plotted as functions of complex *E*. The green dotted circles on the $|\beta _1|$ and $|\beta _2|$ surfaces are the projections of the open-chain PBC eigenenergy spectrum onto the spectrum. The values of (**b**) $|\beta _{1,2}|$, (**c**) $\textrm{Ln}\ |b_{1,2}|$, and (**d**) $|\psi _{\textrm{CHN},(1,N)}|$ for $N=30$, 40, and 50 at the eigenenergies of the respective chain lengths. Parameters used: $t_{\textrm{L}}=2$, $t_{\textrm{R}}=1$, and $\Gamma =10^{-3}$.

The plots in Fig. 3 depict the values of $|\beta _{1,2}|$, $|b_{1,2}|$ and $|\psi _{\textrm{CHN}, (1,N)}|$ on the complex energy plane *E* for three different lengths of the closed loop in the long-chain regime. The values of $|\beta _{1,2}|$ are also plotted across the complex energy plane in Fig. 3a.

Figure 3a shows that within the ellipse of the open-chain PBC eigenenergy loci denoted by the dotted circles, both $|\beta _1|$ and $|\beta _2|$ are greater than 1, and that $|\beta _2| > |\beta _1|$. (By definition, the PBC eigenenergy locus is the locus of the eigenvalues of Eq. (3) for which *k* is real, i.e., $|\beta _1|=1$. The projection of the eigenenergy locus therefore cuts the $|\beta _1|$ surface at $|\beta _1|=1$. ) These facts will be made use of the derivations that follow. Figure 3b shows that as the length of the closed chains increases, the value of $|\beta _1|$ in the eigenstates tends towards the PBC value of 1 while that of $|\beta _2|$ reduces with increasing length. Figure 3c shows that the relative weight of $\beta _2$ to $\beta _1$ decreases as the length of closed chain increases. Taken together, these two trends imply that the eigenstates in the closed chains approach the PBC eigenstate $\psi _{\textrm{PBC}}(n) = \exp (i k n),\ k \in \mathbb {R}$ as the length of the chain increases.

The preceding arguments may give the impression that the NHSE will diminish as the length of the closed chains become longer. Such a claim has in fact been made in a very recent study on a related system^44^. However, Fig. 3d shows that the relative magnitudes between the wavefunctions at $n=1$ and $n=N$ remain fairly constant regardless of the length of the chain. Furthermore, by comparing Figs. 1d and 1e, it can be seen that the localization of the bulk modes remains undiminished in the long-chain regime compared to that in the short-chain regime. This may be qualitatively understood as a consequence of the boundary condition given in Eq. (13): because of the exponential dependence of $\psi _{\textrm{CHN}}(n)$ on *n* and the fact that both $|\beta _1|$ and $|\beta _2|$ are greater than 1, the $\psi _{\textrm{CHN},N}$ term on the left hand side of Eq. (13) tends to be substantially larger than the $E\psi _{\textrm{CHN}, 1} - t_{\textrm{R}}\psi _{\textrm{CHN}}(2)$ terms on the right hand side. Hence, for the boundary condition to still hold, the magnitude of $\psi _{\textrm{CHN},N}$ needs to be moderated. From Eq. (12), $\psi _{\textrm{CHN},N}\approx t_{\textrm{L}}\psi _{\textrm{CNH}}(N-1)$ because $\Gamma \psi _{\textrm{CNH},1}$ is negligibly small compared to the other two terms. The moderation of $|\psi _{\textrm{CHN},N}|$ is accomplished through the mutual cancellation of the terms containing $\beta _1$ and $\beta _2$ in $\psi _{\textrm{CHN}}(N-1)$, which has the form given in Eq. (19). We find numerically that the $b_1|\beta _1|^{N-1}$ and $b_2|\beta _2|^{N-1}$ terms in $\psi _{\textrm{CHN}}(N-1)$ have roughly the same order of magnitudes that do not vary substantially with *N*.

To explain these findings and those in Fig. 3c,d more qualitatively, we turn to a more rigorous derivation of the wavefunction in the long-chain regime. In analogy to the short-chain regime, the expression for $\psi _{\textrm{CHN}}(n)$ in Eq. (19) can be substituted into Eq. (10) and (11) with $b_1$ set as 1. Subsequently, $\psi _{\textrm{CHN}, 1}$ and $\psi _{\textrm{CHN}, N}$ can be solved for in terms of $b_2$. The resulting expressions for $\psi _{\textrm{CHN}, 1}$ and $\psi _{\textrm{CHN}, N}$ are then be substituted into Eq. (12) to solve for $b_2$. We then obtain

20 $$\begin{aligned} b_2 = \frac{ |\beta _1|^2 \exp (i(2+N)k_1)t_{\textrm{R}}(|\beta _1|^N\exp (iNk_1)t_{\textrm{R}} - \Gamma )}{t_{\textrm{l}}( -\exp (iNk_2)|\beta _2|^N t_{\textrm{R}} + \Gamma )} . \end{aligned}$$

Because $\Gamma$ has a much smaller absolute value than $|\beta _1|^N$ in the numerator and $|\beta _2|^N$ in the denominator, $\Gamma$ can be dropped in both the numerator and denominator of the above equation to a good approximation. By considering Eq. (18), Eq. (20) is then approximated as

21 $$\begin{aligned} b_2 \approx -\exp (2i(N+1)k_1)\left( \frac{|\beta _1|}{|\beta _2|}\right) ^{N+1}. \end{aligned}$$

Because $|\beta _1|/|\beta _2| < 1$, as shown in Fig. 3a, Eq. (21) agrees with the numerical results in Fig. 3c that $|b_2|$ decreases exponentially with *N*. Meanwhile, under the same approximation, $|\psi _{\textrm{CHN}, n}|$ is given by

22 $$\begin{aligned} |\psi _{\textrm{CHN}}(n)| \approx |\beta _1|^{n} (1 - |\beta _1|^2\frac{t_r}{t_l}\exp (2ik_1)),\ n=2,\ldots ,N-1 . \end{aligned}$$

Using the approximate value for $|\psi _{\textrm{CHN}}(N-1)|$ given by Eq. (22) and the approximation $\psi _{\textrm{CHN},N}\approx t_{\textrm{L}}\psi _{\textrm{CNH}}(N-1)$, we find that

23 $$\begin{aligned} |\psi _{\textrm{CHN},N}|\approx |\psi _{\textrm{CHN}}(N-1)|. \end{aligned}$$

Substituting the approximation for $b_2$ in Eq. (21) into Eq. (19) and the boundary condition Eq. (13) and using the fact that $|\beta _1|/|\beta _2| < 1$, we obtain the following relation that needs to be satisfied by $|\beta _1|$ in an eigenstate:

24 $$\Gamma |\beta _1|^N \left| 1-\exp (2ik)|\beta _1|^2\frac{t_{\textrm{R}}}{t_{\textrm{L}}} \right| = t_{\textrm{L}}.$$

**Figure 4:**
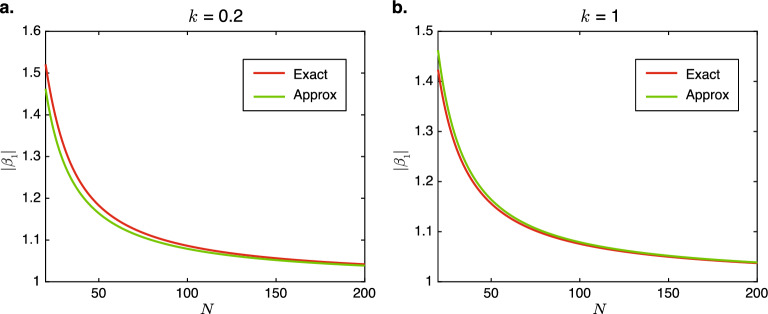
Comparison of the exact solution for $|\beta _1|$ Eq. (24) and approximate solution Eq. (25) for $t_{\textrm{L}} = 2$, $t_{\textrm{R}}=1$, and $\Gamma =10^{-3}$ at (**a**) $k=0.2$, and (**b**) $k=1$.

This, together with Eq. (22), implies that $|\psi _{\textrm{CHN},N}| \approx \frac{t_{\textrm{L}}}{\Gamma }$, which is consistent with the near independence of $|\psi _{\textrm{CHN},N}|$ on *N*, as depicted numerically in Fig. 3d. When $N \gg 2$, this gives the rough approximation that

25 $$\begin{aligned} |\beta _1|^N \approx \frac{1}{\Gamma } t_{\textrm{L}}, \end{aligned}$$

which is consistent with the decrease in $|\beta _1|$ with increasing *N* in Fig. 3b. The adequacy of this approximation is demonstrated with in Fig. 4 where the approximate values of $|\beta _1|$ obtained using Eq. (25) are compared with those computed numerically from Eq. (24).

Interestingly, Eq. (25) implies that the skin effect decay length $1/(\textrm{Ln}\ |\beta _1|)$ relative to the total length of the chain, i.e., $(N\textrm{Ln}\ |\beta _1|)^{-1}$ is a constant. Therefore, similar to the conventional CNHSE in open chains, the skin effect in the long-chain regime is scale-free^41,45^. In other words, the proportion of the chain in which the magnitude of the wavefunction exceeds an arbitrary threshold is independent of the chain length. The scale-free nature of the NHSE can be seen by comparing Figs. 1d and 1e.

### Generality of the TCIEST

**Figure 5:**
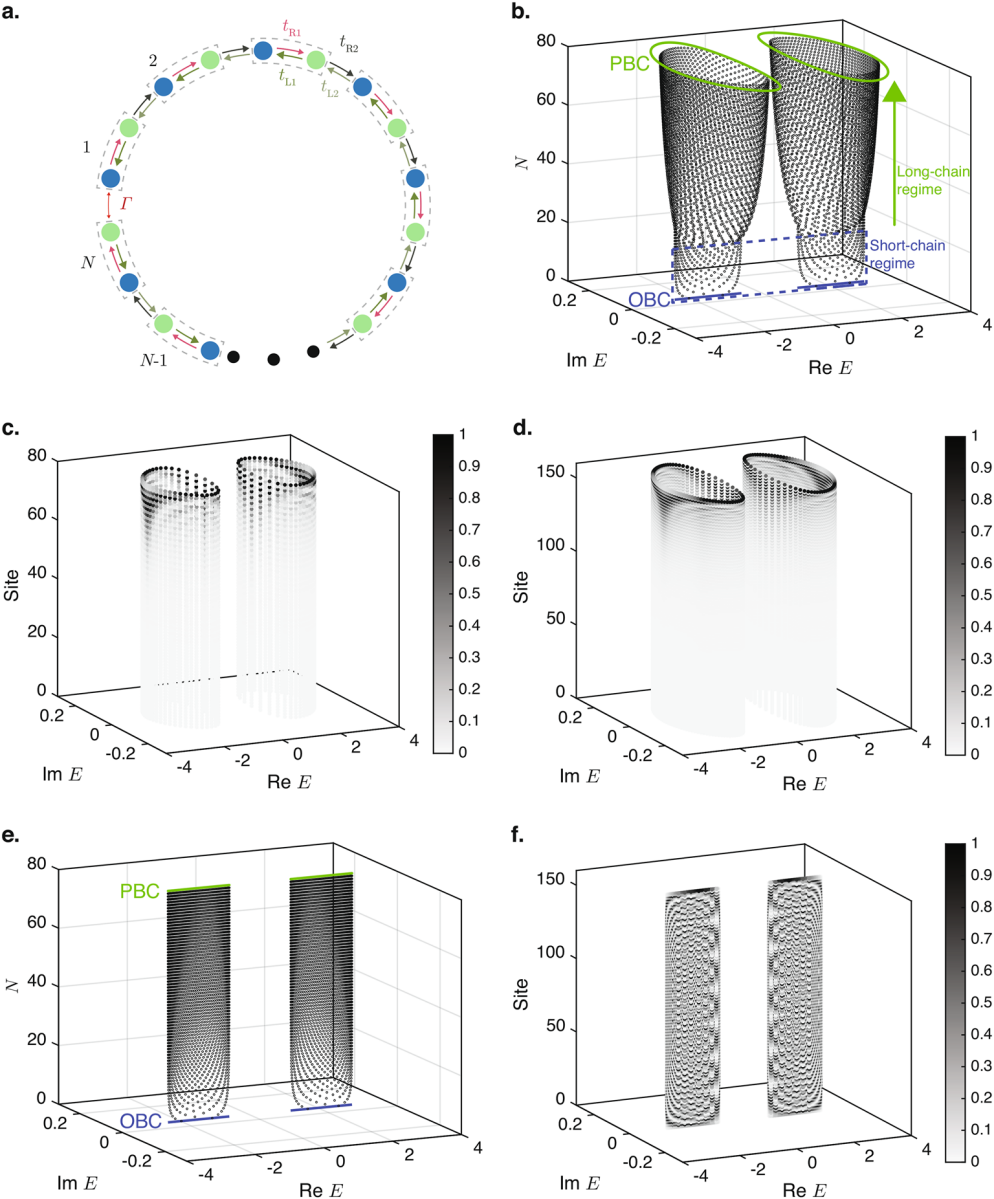
Eigenspectrum and density distribution of closed Su–Schrieffer–Heeger (SSH) loops. (**a**) A schematic representation of a closed SSH chain with *N* unit cells and the intra-unit cell couplings of $t_{\textrm{(L,R)},1}$, inter-unit cell couplings of $t_{\textrm{(L,R)},2}$, and terminal coupling between the first and last node of $\Gamma$. Each unit cell is denoted by a dashed box. (**b**) The energy spectrum of a closed SSH loop with $t_{\textrm{L1}}=2.9$, $t_{\textrm{R1}}=1.1$, $t_{\textrm{L2}}=0.775$, $t_{\textrm{R2}}=1.225$, and $\Gamma =10^{-3}$ as a function of *N*. The open-chain OBC and PBC spectra are also plotted in the figure for comparison. (**c**,**d**) The eigenenergy spectra and density distributions across the lattice sites for the eigenmodes of the closed SSH loop in (**b**) at $N=40$ and $N=80$ respectively. Darker colors indicate higher densities. (Note that the number of sites is twice the number of unit cells because each unit cell contains two sites.) (**e**) The energy spectrum of a closed SSH loop at $\Gamma =10^{-3}$, and the critical parameter values of $t_{\textrm{L1}}=2.9$, $t_{\textrm{L1}}=1.1$, $t_{\textrm{L2}}=0.55$ and $t_{\textrm{R2}}=1.45$. (**f**) The eigenenergy spectrum and density distribution across the lattice sites for the eigenmodes of the critical closed SSH chain in (**e**) for $N=80$.

We now demonstrate the generality of the TCIEST in more complex closed loop systems beyond the closed HN loop. The first example is the non-Hermitian Su-Schrieffer-Heeger (SSH) chain. In contrast to the HN chain in which each unit cell contains only a single node, the unit cell of the SSH chain contains two nodes with the characteristic feature that the inter-cell and intra-cell couplings differ from each other. The Hamiltonian of a closed SSH chain containing *N* unit cells with the intra-unit cell couplings of $t_{(\textrm{L,R}), 1}$ in the left and right directions, inter-unit cell coupling of $t_{\textrm{(L,R)},2}$, and terminal coupling of $\Gamma$ between the first and last sites can be written as

26 $$\begin{aligned} H_{\textrm{SSH}}&= \left( \sum _j^{N} |2j-1\rangle t_{\textrm{R}1} \langle 2j| + |2j\rangle t_{\textrm{L}1} \langle 2j-1|\right) \nonumber \\&\quad + \left( \sum _j^{N-1} |2j\rangle t_{\textrm{R}2} \langle 2j+1| + |2j+1\rangle t_{\textrm{L}2} \langle 2j| \right) \nonumber \\&\quad + \Gamma ( |1\rangle \langle 2N| + |2N\rangle \langle 1|) \end{aligned}$$

where the first row represents the inter-unit cell couplings, the second row the intra-unit cell couplings, and the last row the terminal coupling between the first and last lattice sites. The system represented by the Hamiltonian in Eq. (26) is schematically illustrated in Fig. 5a.

Figure 5b shows the occurrence of the TCIEST for an exemplary parameter set in a SSH chain. A clear signature of the TCIEST can be seen around $N=20$. Here, a transition occurs in the energy spectrum from the open-chain OBC form comprising two separated lines on the real energy axis, which characterizes the short-chain regime, to the open-chain PBC form comprising two ellipses on the complex energy plane symmetrically distributed about the imaginary axis, which characterizes the long-chain regime. Figure 5c,d show the density distributions for two chains with the same parameters as those in Fig. 5b at the lengths of 40 and 80 unit cells, respectively, which fall within the long-chain regime. Note that despite the loop in Fig. 5d being twice as long as that in Fig. 5c, the spatial density distributions of the eigenmodes for both chains relative to the total length are broadly similar. This again demonstrates the scale-free characteristic of the TCIEST effect.

By applying the standard condition that the loci of the eigenenergies in the thermodynamic-limit OBC open-chain energy spectrum is given by energy values at which the middle values of $\beta$ have the same absolute values^43^, the inverse decay skin length of the open-chain SSH system under OBC, $(\textrm{Ln}\ |\beta |)$ can be derived to be

27 $$\begin{aligned} \textrm{Ln}\ |\beta | = \frac{1}{2} \textrm{Ln}\ \left| \frac{t_{\textrm{L}1}t_{\textrm{L}2}}{t_{\textrm{R}1}t_{\textrm{R}2}} \right| . \end{aligned}$$

Eq. (27) implies that there is a critical set of parameters at which the inverse skin length is zero, i.e., there is no localization of the eigenstates near the edges even when the system is non-Hermitian. This occurs when the condition

28 $$\begin{aligned} |t_{\textrm{L}1}t_{\textrm{L}2}| = |t_{\textrm{R}1}t_{\textrm{R}2}| \end{aligned}$$

is satisfied^12^. Figure 5e shows the eigenenergy spectra for a closed SSH chain with an exemplary set of parameters satisfying Eq. (28) along with the PBC and OBC spectra of the corresponding open SSH chains. Both the OBC and PBC spectra of the open chains have the same form, i.e., both consist of two discontinuous lines on the real axis. The correspondence between the two implies that there is no skin effect in the system, as expected. The absence of the NHSE is further demonstrated in the plot of the density distributions of the closed-chain eigenstates at $N=80$ in Fig. 5f, which clearly shows the absence of exponential localization of the eigenmodes near either of the edges. As explained earlier, the transition from the short- to long-chain regime in the TCIEST depends on the wavefunction at one end of the chain being exponentially larger than that at the other end in the NHSE. Thus, the absence of any exponential localization in Fig. 5e implies the absence of TCIEST in this particular closed SSH chain when the coupling parameters satisfy Eq. (28).

### Co-existence of the TCIEST and CNHSE

**Figure 6:**
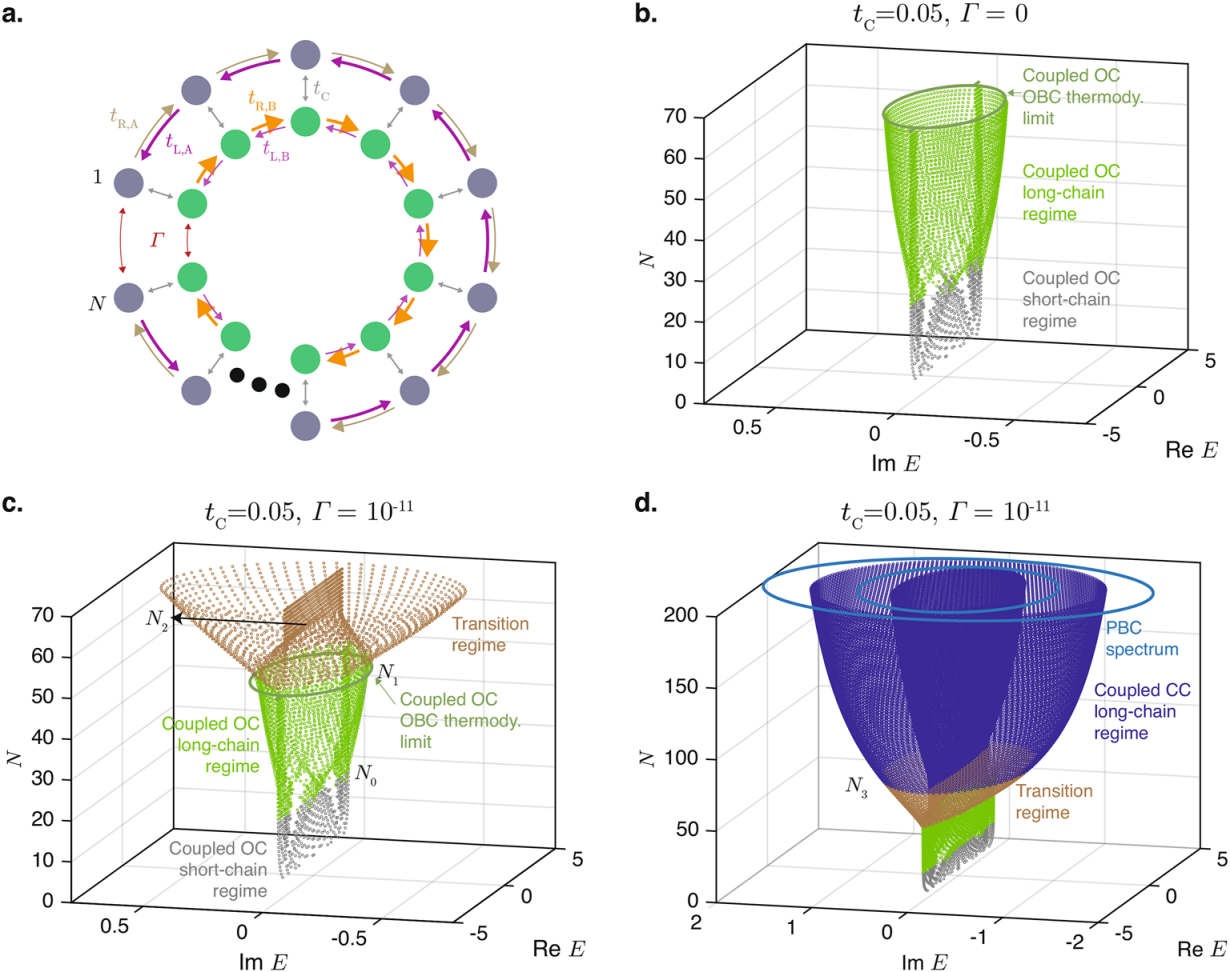
Co-existence of TCIEST and critical non-Hermitian skin effect. (**a**) A schematic representation of two Hatano–Nelson chains each containing *N* nodes and the respective coupling strengths of $t_{\textrm{(L,R)}, A}$ and $t_{\textrm{(L,R)}, B}$. Each node in one chain is coupled to its corresponding node in the other chain by the inter-chain coupling $t_{\textrm{C}}$. (**b**) The energy spectrum of the coupled open chain (“coupled OC”) as a function of the chain length. The OBC spectrum in the thermodynamic limit of the open chain is shown as well. (**c**) The energy spectrum of the coupled closed loop as a function of the chain length. (**d**) The energy spectrum of the same loop in c. as a function of the chain length for longer lengths of the chain and the PBC eigenenergy spectrum. The different regimes in each of these systems are indicated by the colors of the dots. The approximate system sizes $N_0$ to $N_3$ at which transitions between different regimes occur are labelled. Common parameters used: $t_{\textrm{L,A}}=2$, $t_{\textrm{L,B}}=3$, $t_{\textrm{R,A}} = t_{\textrm{R,B}} = 1$, $t_{\textrm{C}}=0.05$, and $\Gamma =10^{-11}$.

We next demonstrate the co-existence of the TCIEST and CNHSE effects in a system comprising two closed HN loops denoted as chains A and B with the respective coupling constants of $t_{(\textrm{L,R}), A}$ and $t_{(\textrm{L,R}), B}$ coupled in parallel. Each site in chain A is coupled to its corresponding site in chain B by a reciprocal coupling $t_{\textrm{C}}$, and the first and last nodes of each chain are coupled by a reciprocal terminal coupling $\Gamma$ to form a closed loop, as shown schematically in Fig. 6a. The Hamiltonian for this system with *N* sites in each chain can be written as

29 $$\begin{aligned} H_{\textrm{PHN}}&= \sum _j^{N-1} \Big ( |j,\textrm{A}\rangle t_{\textrm{R,A}} \langle j+1,\textrm{A}| + |j,\textrm{B}\rangle t_{\textrm{R,B}} \langle j+1,\textrm{B}| \nonumber \\&\quad \times |j+1,\textrm{A}\rangle t_{\textrm{L, A}} \langle j,\textrm{A}| + |j+1,\textrm{B}\rangle t_{\textrm{L, B}} \langle j,\textrm{B}| \Big ) \nonumber \\&\quad + \sum _j^{N} ( |j,\textrm{A}\rangle t_c \langle j,\textrm{B}| + |j,\textrm{B}\rangle t_{\textrm{C}} \langle j,\textrm{A}| ) \nonumber \\&\quad + \Gamma ( |1,\textrm{A}\rangle \langle N,\textrm{A}| + |1,\textrm{B}\rangle \langle N,\textrm{B}|). \end{aligned}$$

Figure 6b shows the eigenenergy spectrum of the system in which $\Gamma$ was set to zero while $t_{\textrm{C}}$ has a finite value. This corresponds effectively to two open-chain NH systems that are coupled to each other in parallel. Such a system exhibits the CNHSE where the eigenenergy spectrum switches from the short-chain regime, which consists of a line on the real axis formed from the union of the OBC spectra of the two individual chains, to the long-chain regime. The switching occurs at a critical chain length *N* of around 20. Figure 6c shows the eigenenergy spectrum of the coupled closed chain system where the terminal coupling $\Gamma$ is now set to a small but finite value of $10^{-11}$. The same transition of the eigenenergy spectrum from the straight line of the open-chain OBC spectra to the elliptical form of the coupled open chain occurs at around the same critical value of $N=N_0$, which is approximately given by

30 $$\begin{aligned} N_0 \approx 4 \frac{\textrm{Ln} (t_{\textrm{C}})}{ \textrm{Ln} \frac{t_{\textrm{L,A}}t_{\textrm{R,B}}}{t_{\textrm{R,A}}t_{\textrm{L,B}}} }, \end{aligned}$$

which we derive in detail in the Methods section. (The exact value of $N_0$ at each eigenenergy and the other critical values $N_1$ to $N_3$ dicusssed later varies slightly with the energy.) The presence of only the interchain coupling $t_{\textrm{C}}$ but not the terminal coupling $\Gamma$ in Eq. (30) indicates that this transition at $N=N_0$ is driven by the CNHSE and not the TCIEST. Interestingly, a finite terminal $\Gamma$ coupling that transforms the NH chains into a closed loop induces a second transition at the critical size $N_1$, which falls around 15 and is approximately given by

31 $$\begin{aligned} N_1 \approx -2\frac{\textrm{Ln}\Gamma }{\textrm{Ln}\frac{t_{\textrm{L,B}}}{t_{\textrm{R,B}}}}. \end{aligned}$$

At this critical point, the eigenenergy spectrum begins to transit from a single ellipse to an intermediate form comprising a straight line enclosed within a larger ellipse. This transition is completed at the crticial size of

32 $$\begin{aligned} N_2 \approx \frac{2\textrm{Ln}\left( \frac{t_{\textrm{C}}^2}{\Gamma }\right) }{\textrm{Ln} \frac{t_{\textrm{L,A}}}{t_{\textrm{R,A}}}}. \end{aligned}$$

The ellipse corresponds to the PBC spectrum of chain A, which has transited to the long-chain regime because of its shorter TCIEST critical length, whereas the straight line corresponds to the OBC spectrum of chain B, which has a longer TCIEST critical length and thus is still within the TCIEST short-chain regime. Figure 6d shows the eigenenergy spectrum of the same system as Fig. 6c for a larger range of *N*. The eigenenergy spectrum undergoes another transition when the energy spectra of the states associated with chain B also make the TCIEST transition at the critical energy $N_3$, which has an approximate value of 75 and is approximately given by

33 $$\begin{aligned} N_3 -\frac{\textrm{Ln}\Gamma }{\textrm{Ln} \sqrt{\frac{t_{\textrm{L,A}}}{t_{\textrm{R,A}}}}} . \end{aligned}$$

The spectrum of these states transforms from the straight line OBC form to the elliptical PBC form.

To confirm our identification of the various size regimes in Fig. 6, we analyze the wavefunction composition of the eigenstates in more detail. In an analogous approach to Eq. (19), the wavefunction within the composite system consisting of two HN chains of length *N* coupled together in parallel can be written in the form of

34 $$\begin{aligned} \psi _{\textrm{PHN}}(x) = \sum _j^4 |\phi _j\rangle b_j \beta _j^x,\ 1< x < N, |\beta _1|\le |\beta _2| \le |\beta _3| \le |\beta _4| \end{aligned}$$

where the $|\phi _j\rangle$s are the eigenspinors of $H_{\textrm{PHN}}(\beta )$ with the eigenenergy *E*, i.e.,

35 $$\begin{aligned} H_{\textrm{PHN}}(\beta _j)|\phi _j\rangle&= |\phi _j\rangle E \end{aligned}$$

36 $$\begin{aligned} H_{\textrm{PHN}}(\beta )&= \begin{pmatrix} t_{\textrm{R,A}}\beta + t_{\textrm{L,A}}/\beta &{} t_{\textrm{C}} \\ t_{\textrm{C}} &{} t_{\textrm{R,B}}\beta + t_{\textrm{L,B}}/\beta \end{pmatrix} . \end{aligned}$$

**Figure 7:**
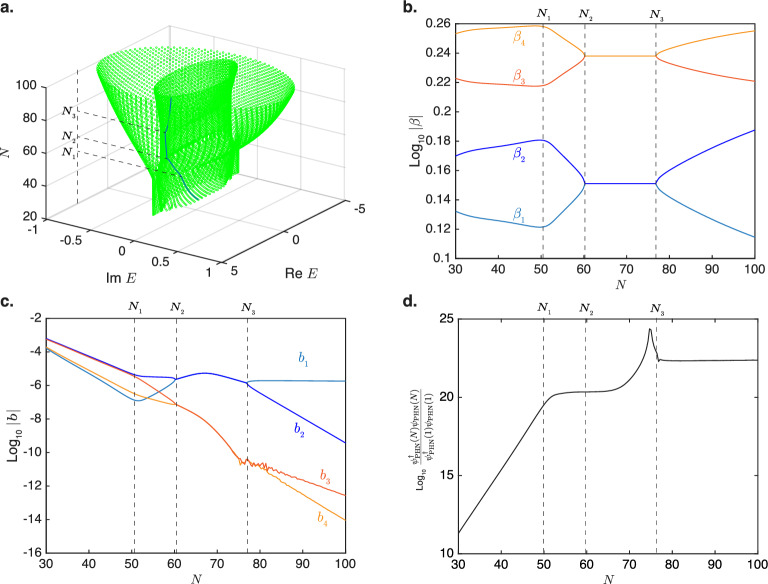
Evolution of eigenstate wavefunction with increasing size. (**a**) The thick blue line traces the eigenenergy trajectory of the eigenstate used for illustration as *N* is varied continuously from 30 to 100 for the model parameters shown in Fig. 6. $N_1$, $N_2$, and $N_3$ denote critical system sizes at which the system switches between the various regimes. (**b**) Variation of the $|\beta _i|$s with *N* on the trajectory shown in panel (**a**). (**c**) Variation of the $|b_i|$s with *N* on the trajectory shown in panel (**a**). (**d**) Variation of $(|\psi _{\textrm{PHN}}^\dagger (N)\psi _{\textrm{PHN}}(N))/ (\psi _{\textrm{PHN}}^\dagger (1)\psi _{\textrm{PHN}}(1))|$ with *N* on the trajectory shown in panel (**a**).

Applying the time-indepndent Schrödinger equation $H_{\textrm{PHN}}\psi _{\textrm{PHN}}(x) = E \psi _{\textrm{PHN}}(x)$ at the two ends of the chain at $x=1$ and $x=N$ gives the boundary conditions

37 $$\begin{aligned} \begin{pmatrix} 0 &{} t_{\textrm{C}} \\ t_{\textrm{C}} &{} 0 \end{pmatrix} \psi _{\textrm{PHN}}(N) - E \psi _{\textrm{PHN}}(N) + \begin{pmatrix} t_{\textrm{L,A}} &{} 0 \\ 0 &{} t_{\textrm{L,B}} \end{pmatrix} \psi _{\textrm{PHN}}(N-1)&= -\Gamma \psi _{\textrm{PHN}}(1), \end{aligned}$$

38 $$\begin{aligned} \begin{pmatrix} 0 &{} t_{\textrm{C}} \\ t_{\textrm{C}} &{} 0 \end{pmatrix} \psi _{\textrm{PHN}}(1) - E \psi _{\textrm{PHN}}(1) + \begin{pmatrix} t_{\textrm{R,A}} &{} 0 \\ 0 &{} t_{\textrm{R,B}} \end{pmatrix} \psi _{\textrm{PHN}}(2)&= -\Gamma \psi _{\textrm{PHN}}(N) . \end{aligned}$$

To investigate how the wavefunction $\psi _{\textrm{PHN}}$ evolves as the size of the system increases, we treat *N* as a continuous variable and study how the $\beta _i$s and $b_i$s in Eq. (34) change as *N* is varied continuously via the following procedure: we first set *E* to one of the eigenenergy values at the integer value of $N=20$, as shown in Fig. 7. We then shift *N* by a small amount and find the nearest value of *E* to the original eigenenergy that satisfies Eq. (35) to (38). By doing this repeatedly, we obtain the continuous trajectory for the eigenenergy of *E* shown in Fig. 7a as *N* is varied continuously. It should be noted that this trajectory indeed cuts across the eigenenergies of *E* computed numerically by the direct diagonalization of Eq. (29) at integer values of *N*. Note that whereas the values of $N_1$ to $N_3$ mentioned earlier are indicative values for the transitions between different regimes across the various eigenstates with different energies here , the numerical values listed in the following discussion in the following are exact values for the specific sequence of eigenstates along the trajectory shown in Fig. 7a.

For $N=20$ to $N=N_1=49.9$, the eigenenergy spectrum of the system tends towards the thermodynamic-limit GBZ of the two coupled chains without the terminal coupling. Besides the visual similarity of the eigenenergy spectrum to that of the GBZ spectrum, as shown in Fig. 6b,c, the fact that the eigenergy spectrum tends towards the GBZ spectrum can also be ascertained from the fact that for $20<N<N_1$, the moduli of the two middle $\beta$ value (i.e., $\beta _2$ and $\beta _3$) converge towards each other as *N* increases, as shown in Fig. 7b. The large moduli of their corresponding weights $b_2$ and $b_3$ in the wavefunction in this range of *N* shown in Fig. 7c show that the $\beta _2$ and $\beta _3$ states are the dominant components in the wavefunction. This convergence of the middle $\beta$ values towards each other as the system size increases is a characteristic feature of systems exhibiting the NHSE under OBC. As we explained in detail in our earlier work^11^, this convergence ensures that the wavefunction components can consistently cancel one another out at both open ends of the system in order to fulfill the OBC that the wavefunctions must vanish at the two open ends. This boundary condition for an open system is equivalent to setting $\Gamma =0$ on the right hand sides of Eqs. (37) and (38).

We now consider the boundary conditions Eqs. (37) and (38) at finite values of $\Gamma$. Figure 7b shows that all the $\beta$ values have moduli larger than 1. This implies that the wavefunctions at $x=N-1$ and $x=N$ have much larger amplitudes than those at $x=1$ and $x=2$, and that the disparity between the magnitudes of the wavefunctions at the two ends of the chain increases with the size of the system. As a result, the very much smaller amplitude of $\Gamma \psi _{\textrm{PHN}}(1)$ on the right side of Eq. (37) compared to those of the terms on the left side means that the terms on the right side of Eq. (37) can be approximated as 0. Meanwhile, for small system sizes at which the disparities between the magnitudes of $\psi _{\textrm{PHN}}(N)$ and $\psi _{\textrm{PHN}}(1)$ is below some critical value, the $\Gamma \psi _{\textrm{PHN}}(N)$ term on the right hand side of Eq. (38) can also be approximated as 0 because of the very small magnitude of $\Gamma =10^{-11}$. In this case where the approximation of taking the right hand sides of Eqs. (37) and (38) to be 0 holds, Eqs. (37) and (38) for the chain with the $\Gamma$ coupling are approximately the same boundary conditions for an open chain without the terminal coupling. The eigenenergy spectrum of the chain with the terminal coupling therefore resembles that of the open chain.

As the system size increases gradually from $N=20$ to $N=N_1$, the disparity between the wavefunction amplitudes at $\psi _{\textrm{PHN}}(1)$ and $\psi _{\textrm{PHN}}(N)$ increases because of the exponential growth of the wavefunction amplitude across the length of the system over larger lengths of the system. The magnitude of the $-\Gamma \psi _{\textrm{PHN}}(N)$ on the right side of Eq. (38) relative to the other terms therefore increases with the system length as shown in Fig. 7d, which shows the ratio of the magnitudes of $\psi _{\textrm{PHN}}(N)$ to $\Gamma \psi _{\textrm{PHN}}(1)$. When the system length increases to the critical value of $N=N_1$, the approximation of taking the right hand side of Eq. (38) to be approximately 0 breaks down, and the eigenenergy spectrum of the system begins to deviate from that of the system without the terminal coupling. The presence of $\Gamma$ in the approximate expression for $N_1$ in Eq. (31) indicates that this transition is driven by the TCIEST.

The transition at $N=N_1$ is similar to the discussion immediately following Eq. (13) for the HN chain where in both cases, the exponential growth of the disparities between the wavefunction amplitudes at the two ends with increasing system size makes it impossible to satisfy the boundary conditions imposed by the end-to-end terminal coupling under the original OBC eigenenergy spectrum as the system size increases beyond a critical value. As a result, the system enters into a transitory regime for system sizes between $N_1$ and $N_2=60.4$ as the eigenenergy spectrum transits from one resembling the OBC GBZ of the two coupled chains to one that resembles the union of the PBC spectrum of one component chain and the OBC GBZ of the other component chain. The presence of both $t_{\textrm{C}}$, which gives rise to the CNHSE, and $\Gamma$, which gives rise to the TCIEST, in the approximate expression for $N_2$ in Eq. (32) indicates that the transition at $N_2$ is due to the interplay of the TCIEST and the CNHSE. Our chosen trajectory in Fig. 7a follows the OBC GBZ of Chain A, as can be seen from the eigenenergy values lie on the real energy axis and that the two main constituent components of the wavefunction ($b_1$ and $b_2$ in Fig. 7c) have $\beta$ values with almost the same moduli but are not the middle $\beta$ values. This resembles the scenario of a system composed of two HN chains with dissimilar decay lengths coupled in parallel (without the terminal end-to-end coupling) where, as explained in detail in our earlier work^11^, at system sizes smaller than the CNHSE transition, the eigenenergy spectrum falls on the real line on which $|\beta _1|=|\beta _2|$ and $|\beta _3|=|\beta _4|$ and the wavefunction is composed primarily of either the $\beta _1$ and $\beta _2$ states or the $\beta _3$ and $\beta _4$ states. We speculate that such a configuration of the eigenstate wavefunction that is also adopted in the small-size limit of two coupled chains without the terminal coupling can also be explained by the right hand side of Eq. (38) being sufficiently close to 0 compared to the other terms at this stage that the system with end-to-end terminal coupling adopts a similar eigenenergy distribution to the one without end-to-end coupling for some of the eigenstates. This hypothesis is supported by the fact that at values of *N* slightly larger than $N_2$, the ratio of the amplitude of $\psi _{\textrm{PHN}}(N)$ to that of $\psi _{\textrm{PHN}}(1)$ grows at a relatively slow rate at *N* increases.

However, as *N* increases further and approaches $N_3=77.0$, the amplitude ratio of the wavefunctions at the two ends starts to increase rapidly again and the approximation of taking the right hand side of Eq. (38) as 0 breaks down again. This results in the eigenenergy spectrum transiting to reach that of the PBC spectrum beyond $N=N_3$, as can be seen from the fact that the wavefunction becomes increasingly dominated by the $b_1$ state (the magnitudes of $b_2$, $b_3$, and $b_4$ relative to that of $b_1$ drop rapidly in Fig. 7c), for which its corresponding $|\beta _1|$ approaches the PBC value of 1 (i.e., $\textrm{log}_{10} |\beta _1| \rightarrow 0$ in Fig. 7b) as *N* increases. The fact that the transition at $N_3$ is driven by the TCIEST of chain A can be seen from the presence of $\Gamma$ and $t_{(\mathrm {L/R})A}$ in the approximate expression for $N_3$ in Eq. (33).

## Discussion

In summary, we reported the terminal-coupling-induced eigenergy spectrum (TCIEST), which, similar to the CNHSE, results a critical transition of the thermodynamic-limit energy spectrum even for an infinitesimally small coupling in which there is a sharp transition between the short-length and long-length regimes of the eigenenergy spectra when the system size increases beyond a coupling-strength dependent critical length. The TCIEST differs from the CNHSE in two key aspects: (1) the CNHSE arises when two non-Hermitian chains with different non-Hermitian skin lengths are coupled together in parallel. In contrast, the TCIEST arises simply by coupling the two ends of a single non-Hermitian chain. (2) In the CNHSE, the eigenenergy spectrum of the coupled system undergoes a switch from the union of the OBC spectra of the individual constituent chains to the OBC spectrum of the coupled system when the system size exceeds the critical length. In the TCIEST case, the eigenenergy spectrum transitions from the open-chain OBC spectrum to the PBC spectrum. Despite these differences, the TCIEST shares the common feature of scale-free localization with conventional CNHSE. The TCIEST phenomenon is general. We demonstrated its occurrence in the SSH loop as well as in a system where two HN chains are coupled together in parallel. In the former, the TCIEST vanishes when the coupling constants are tuned to values where the NHSE is suppressed in open SSH chains. In the latter, we demonstrate the co-existence of the TCIEST and CNHSE and the resulting multiple transitions of the eigenenergy spectrum as the system length is varied. The ubiquity of the TCIEST effect in various non-Hermitian systems coupled with the sharp and robust changes of their eigenenergy spectra may be utilized for potential applications such as electrical switching, sensing, and multiple-state data storage.

**Figure 8:**
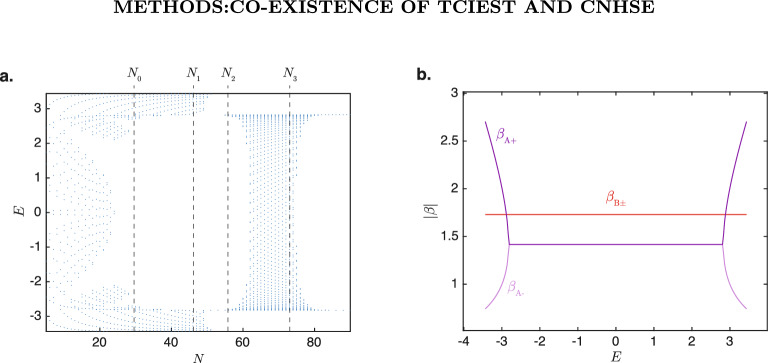
Real eigenenergies in parallel-coupled NH chains with terminal coupling. (**a**) Real eigenvalues *E* of system of parallel-coupled NH chains with terminal coupling in Fig. 6a at size *N*. The values of the critical system sizes $N_0$ to $N_3$ obtained from Eqs. (30) to (33) are indicated. (**b**) The moduli of $\beta _{(\textrm{A,B}),\pm }$ for system in (**a**) across the range of real eigenenergies *E* considered.

We derive approximate analytic expressions for $N_0$, $N_1$, $N_2$, and $N_3$ in the system of parallel-coupled NH chains with terminal coupling in Fig. 6a by investigating how its eigenenergies with real values vary with the system size *N*. This variation and the definitions of $N_0$ to $N_3$ are shown in Fig. 8a. To this end, we first derive expressions for $\beta _j$ and $|\phi _j\rangle$s in Eq. (34) and (36). For a given eigenenergy *E*, the characteristic equation $|H_{\textrm{PHN}} - E\textrm{I}| = 0$ gives

39 $$\begin{aligned} t_{\textrm{L,A}}t_{\textrm{L,B}} - E(t_{\textrm{L,A}}+t_{\textrm{L,B}})\beta + (E^2 - t_{\textrm{C}}^2 + t_{\textrm{L,A}}t_{\textrm{R,B}} + t_{\textrm{L,B}}t_{\textrm{R,A}})\beta ^2 - E (t_{\textrm{R,A}}+t_{\textrm{R,A}})\beta ^3 + t_{\textrm{R,A}}t_{\textrm{R,B}}\beta ^4 = 0. \end{aligned}$$

The characteristic equation is, in general, a fourth-order polynomial in $\beta$ for which no simple analytic solution exists. However, when $t_{\textrm{C}}$ is small compared to the $t_{\mathrm {(L,R)},\mathrm {(A,B)}}s$, as in the case here, it is an adequate approximation to retain only the first order in $t_{\textrm{C}}$. Neglecting the $t_{\textrm{C}}^2$ term in Eq. (39), we find that (39) can then be factorized into two decoupled equations for chains A and B:

40 $$\begin{aligned} \left( t_{\textrm{L,A}} + \beta (-E + t_{\textrm{R, A}}\beta )\right) \left( t_{\textrm{L,B}} + \beta (-E + t_{\textrm{R, B}}\beta )\right) = 0, \end{aligned}$$

from which we obtain

41 $$\begin{aligned} \beta _{\textrm{A},\pm } = \frac{E \pm \sqrt{E^2 - 2 t_{\textrm{L,A}}t_{\textrm{R,A}}}}{2\mathrm {t_{\textrm{R, A}}}}, \beta _{\textrm{B}, \pm } = \frac{E \pm \sqrt{E^2 - 2 t_{\textrm{L,B}}t_{\textrm{R,B}}}}{2\mathrm {t_{\textrm{R, B}}}}. \end{aligned}$$

Notice that the $\beta _{\textrm{A},\pm }$s ($\beta _{\textrm{B},\pm }$) form a complex conjugate pair with $|\beta _{\textrm{A}}| = \sqrt{ t_{\textrm{L,A}}/t_{\textrm{R,A}}}$ ($|\beta _{\textrm{B}}| = \sqrt{ t_{\textrm{L,B}}/t_{\textrm{R,B}}}$) , and hence lie on the complex plane GBZ of the uncoupled chain when *E* is real and $E^2 < 2 t_{\textrm{L,A}}t_{\textrm{R,A}}$ ($E^2 < 2 t_{\textrm{L,B}}t_{\textrm{R,B}}$). For our parameter set of $t_{\textrm{L,A}} = 2$, $t_{\textrm{L,B}} = 3$, and $t_{\textrm{R,A}} = t_{\textrm{R,B}} = 1$, both $\beta _{\textrm{A},\pm }$ and $\beta _{\textrm{B},\pm }$ lie on the GBZ of their respective uncoupled chains when $|E| < 2\sqrt{t_{\textrm{L,A}}t_{\textrm{R,A}}}$. Within this energy range, we write $\beta _{\mathrm {(A,B)}\pm } = |\beta _{\mathrm {(A,B)}}|\exp ( \pm i k_{\textrm{A,B}} )$ where $k_{\mathrm {(A,B)}}$ is real.

Because $|t_{\textrm{C}}|$ is small, we expect the $|\phi _j\rangle$s in Eq. (34) corresponding to $\beta = \beta _{\textrm{A},\pm }$ ($\beta = \beta _{\textrm{B},\pm }$) to be largely localized on the A (B) chain with only a small component dependent on $t_{\textrm{C}}$ on the other chain. We therefore write the $|\phi _j\rangle$ corresponding to $\beta = \beta _{\textrm{A},\pm }$ ($\beta = \beta _{\textrm{B},\pm }$) as $(1, \chi _{\mathrm {A,\pm }})^{\textrm{T}}$ [$(\chi _{\mathrm {B,\pm }}, 1)^{\textrm{T}}$] where, to first order in $t_{\textrm{C}}$,

42 $$\begin{aligned} \chi _{\mathrm {(A, B)}\pm } = -t_{\textrm{C}} \frac{\beta _{\mathrm {(A,B)},\pm }}{t_{\mathrm {L,(A,B)}} + t_{\mathrm {R,(A,B)}}\beta _{\mathrm {(A,B)}\pm }^2}. \end{aligned}$$

We write an eigenstate at real *E* for $|E| < 2\sqrt{t_{\textrm{L,A}}t_{\textrm{L,B}}}$ as

43 $$\begin{aligned} \psi _{\textrm{PHN}}(x) =&|\beta _{\textrm{A}}|^x \left( b_{\textrm{A}+} \exp (i k_{\textrm{A}} x) \begin{pmatrix} 1 \\ \chi _{\textrm{A}+} \end{pmatrix} + b_{\textrm{A}-} \exp (-i k_{\textrm{A}} x) \begin{pmatrix} 1 \\ \chi _{\textrm{A}-} \end{pmatrix} \right) \nonumber \\&+ |\beta _{\textrm{B}}|^x \left( b_{\textrm{B}+} \exp (i k_{\textrm{B}} x) \begin{pmatrix} \chi _{\textrm{B}+} \\ 1 \end{pmatrix} + b_{\textrm{B}-} \exp (-i k_{\textrm{B}} x) \begin{pmatrix} \chi _{\textrm{B}-} \\ 1 \end{pmatrix} \right) , \end{aligned}$$

in the boundary conditions Eqs. (37) and (38) where the $b_{(\textrm{A,B}),\pm }$s are unknown coefficients to be determined. The leading terms in the determinant $D_{|E| < 2\sqrt{t_{\textrm{L,A}}t_{\textrm{L,B}}}}$ of the resulting set of linear equations in the $b_{(\textrm{A,B}),\pm }$s then have the form of

44 $$\begin{aligned} D_{|E| < 2\sqrt{t_{\textrm{L,A}}t_{\textrm{L,B}}}}&= \left[ |\beta _{\textrm{B}}|^{2N} t_{\textrm{C}}^2 c_{0,2,0,2} + |\beta _{\textrm{A}}|^N|\beta _{\textrm{B}}|^N ( c_{0,1,1,0} + c_{0,1,1,2} t_{\textrm{C}}^2 + c_{0,1,1,4} t_{\textrm{C}}^4) + |\beta _{\textrm{B}}|^{2N} c_{0,0,2,2} t_{\textrm{C}}^2 \right] \nonumber \\&\quad + \Gamma \left[ |\beta _{\textrm{A}}|^{N}|\beta _{\textrm{B}}|^{2N} ( c_{1,1,2,0} + c_{1,1,2,2}t_{\textrm{C}}^2 + c_{1,1,2,2}t_{\textrm{C}}^4 ) + |\beta _{\textrm{A}}|^{2N}|\beta _{\textrm{B}}|^{N} ( \ldots ) + |\beta _{\textrm{B}}|^N(\ldots ) + |\beta _{\textrm{A}}|^N(\ldots ) \right] \nonumber \\&\quad + \Gamma ^2 \left[ |\beta _{\textrm{A}}|^{2N}||\beta _{\textrm{B}}|^{2N}(c_{2,2,2,0} + c_{2,2,2,2}t_{\textrm{C}}^2) + c_{1,1,2,0} + c_{1,1,2,4}t_{\textrm{C}}^4) + |\beta _{\textrm{B}}|^{2N}(\ldots ) + |\beta _{\textrm{A}}|^{2N}(\ldots ) + |\beta _{\textrm{A}}|^N|\beta _{\textrm{B}}|^N(\ldots ) + \cdots \right] \nonumber \\&\quad + \Gamma ^3 \left[ \left( |\beta _{\textrm{A}}|^N|\beta _{\textrm{B}}|^{2N}(\ldots ) + \cdots \right) \right] + \Gamma ^4 \left[ \left( |\beta _{\textrm{B}}|^{2N}(\ldots ) + |\beta _{\textrm{A}}|^N(\ldots ) + \cdots \right) \right] . \end{aligned}$$

The values of *E* at which $D_{|E| < 2\sqrt{t_{\textrm{L,A}}t_{\textrm{L,B}}}} = 0$ correspond to the eigenenergies of the system. In Eq. (44), the $c_{p_\Gamma , p_{|\beta _{\textrm{A}}|}, p_{|\beta _{\textrm{B}}|}, p_{t_{\textrm{C}}}}$s are the coefficients of the terms containing $\Gamma ^{p_\Gamma }$, $|\beta _{\mathrm {(A/B)}}|^{p_{|\beta _{\mathrm {(A/B)}}|}}$, and $t_{\textrm{C}}^{p_{t_{\textrm{C}}}}$. The $c_{p_\Gamma , p_{|\beta _{\textrm{A}}|}, p_{|\beta _{\textrm{B}}|}, p_{t_{\textrm{C}}}}$s are independent on *N* and, in general, contain exponents of $ik_{(\textrm{A},\textrm{B})}$ and sums and products of the $t_{\mathrm {(A,B),(L,R}}s$. For instance,

45 $$\begin{aligned} t_{\textrm{C}}^2 c_{0,2,0,2}&=|\beta _{\textrm{B}}|^2 t_{\textrm{L,A}}t_{\textrm{L,B}}t_{\textrm{R,A}}t_{\textrm{R,B}}\textrm{Re}( (\chi _{\textrm{A}+} - \chi _{\textrm{A}-})\chi _{\textrm{B}+}), \end{aligned}$$

46 $$\begin{aligned} c_{0,1,1,0}&= 2|\beta _{\textrm{A}}||\beta _{\textrm{B}}| (\cos ((k_1-k_2)(N+1)) - \cos (k_1+k_2)(N+1)),\end{aligned}$$

47 $$\begin{aligned} t_{\textrm{C}}^4 c_{1,1,2,4}&= 2|\beta _{\textrm{A}}|^2t_{\textrm{R,A}}t_{\textrm{R,B}} |\chi _{\textrm{A}+}|^2|\chi _{\textrm{B}+}|^2 (\cos (k_1(1+N)+k_2) - \cos (k_1(N+1)-k_2)). \end{aligned}$$

The explicit expressions for the remaining $c_{p_\Gamma , p_{|\beta _{\textrm{A}}|}, p_{|\beta _{\textrm{B}}|}}$s and the omitted terms in $D_{|E| < 2\sqrt{t_{\textrm{L,A}}t_{\textrm{L,B}}}}$ can be read off the full expressions for $D_{|E| < 2\sqrt{t_{\textrm{L,A}}t_{\textrm{L,B}}}}$ given in Eqs. (54) to (59), which we relegate to the end of this section to avoid disrupting the flow of the argument because these expressions are extremely long. In general, the values of the $c_{p_\Gamma , p_{|\beta _{\textrm{A}}|}, p_{|\beta _{\textrm{B}}|}}$s terms oscillate with the variation of *E* because of the dependence of $k_{\textrm{A,B}}$ and $\chi _{(\textrm{A,B})\pm }$ on $\beta _{(\textrm{A,B}),\pm }$ via Eqs. (41) and (42). Because the magnitudes of the $t_{\mathrm {(L,R)},\mathrm {(A,B)}}$s and $|\beta _{\textrm{A,B}}|$s are all on the order of 1, we adopt the approximation that the maximum magnitudes of all the $c_{p_\Gamma , p_{|\beta _{\textrm{A}}|}, p_{|\beta _{\textrm{B}}|}}$s as they vary with *E* approximately fall within the same order of magnitude.

For our parameter set, $\Gamma =10^{-11}$ is by far the parameter with the smallest magnitude out of $(t_{\mathrm {(L/R)}, \mathrm {(A/B)}}, t_{\textrm{C}}, \Gamma )$, and $1< |\beta _{\textrm{A}}| < |\beta _{\textrm{B}}|$ when $|E| < 2\sqrt{t_{\textrm{L,A}}t_{\textrm{R,A}}}$ (Fig. 8b). The terms in Eq. (44) are therefore arranged in increasing order of $p_\Gamma$, and within each value of $p_\Gamma$, in descending magnitudes of $|\beta _{\textrm{A}}|^{p_{\beta _{\textrm{A}}}}|\beta _{\textrm{B}}|^{p_{\beta _{\textrm{B}}}}$. (The omitted terms in Eq. (44) are all associated with higher powers of $\Gamma$ and / or smaller values of $p_{\beta _{(\textrm{A,B})}}$ than those shown explicitly.) Notice that the leading factor of $|\beta _{\textrm{A}}|^{p_{\beta _{\textrm{A}}}}|\beta _{\textrm{B}}|^{p_{\beta _{\textrm{B}}}}$ in the terms independent of $\Gamma$ in the first row of Eq. (44) is $|\beta _{\textrm{B}}|^{2N}$, while those in the terms associated with $\Gamma$ and $\Gamma ^2$ in the second and third have additional factors of $|\beta _{\textrm{A}}|^N$ and $|\beta _{\textrm{A}}|^{2N}$, respectively.

At small values of *N* at which $|\beta _{\textrm{A}}|^N \Gamma \ll 1$, the dominant contribution to $D_{|E| < 2\sqrt{t_{\textrm{L,A}}t_{\textrm{L,B}}}}$ come from the terms independent of $\Gamma$ in the first row of Eq. (44). The eigenenergy spectrum is therefore primarily determined by the terms independent of $\Gamma$ at small system sizes because the terms containing finite powers of $\Gamma$ are, in general, too small in magnitude to cancel off the terms independent of $\Gamma$ to make the determinant 0. In other words, at small system sizes, the system behaves as if the terminal coupling $\Gamma$ is absent. Among the terms independent of $\Gamma$ in the first row of Eq. (44), the two dominant terms are $|\beta _{\textrm{B}}|^{2N} t_{\textrm{C}}^2 c_{0,2,0,2} + |\beta _{\textrm{A}}|^N|\beta _{\textrm{B}}|^N c_{0,1,1,0}$. Apart from the *c* factors, the first term differs from the second term by a factor of $|\beta _{\textrm{A}}/\beta _{\textrm{B}}|^N t_c^2$ where $|\beta _{\textrm{A}}/\beta _{\textrm{B}}| > 1$ in our parameter set. At small values of *N* at which the magnitude of $|\beta _{\textrm{A}}/\beta _{\textrm{B}}|^N t_{\textrm{C}}^2$ is on the order of or smaller than one, it is possible for the two terms in $|\beta _{\textrm{B}}|^{2N} t_{\textrm{C}}^2 c_{0,2,0,2} + |\beta _{\textrm{A}}|^N|\beta _{\textrm{B}}|^N c_{0,1,1,0}$ to cancel off each other so that $D_{|E| < 2\sqrt{t_{\textrm{L,A}}t_{\textrm{L,B}}}}$ becomes 0, and real eigenvalues with $|E| < 2\sqrt{t_{\textrm{L,A}}t_{\textrm{L,B}}}$ exist. In contrast, when *N* is substantially larger than the critical size

48 $$\begin{aligned} N_0 \simeq -\frac{\textrm{Ln} (t_{\textrm{C}}^2)}{ \textrm{Ln} \frac{|\beta _{\textrm{A}}|}{|\beta _{\textrm{B}}|} } \approx 4 \frac{\textrm{Ln} (t_{\textrm{C}})}{ \textrm{Ln} \frac{t_{\textrm{L,A}}t_{\textrm{R,B}}}{t_{\textrm{R,A}}t_{\textrm{L,B}}} }, \end{aligned}$$

the $|\beta _{\textrm{B}}|^{2N} t_{\textrm{C}}^2 c_{0,2,0,2}$ term becomes overwhelmingly large compared to the $|\beta _{\textrm{A}}|^N|\beta _{\textrm{B}}|^N c_{0,1,1,0}$ term and can no longer be canceled off by the former. As a result, there are no longer real values of eigenenergies *E* with $|E| < 2\sqrt{t_{\textrm{L,A}}t_{\textrm{L,B}}}$ until the critical transition energy $N_2$. The disappearance of real eigenenergies at $N > N_0$ and the concomitant transition of the eigenenergy spectrum from the complex energy GBZ of the isolated chain A in the form of a straight line on the real axis to that of the GBZ of the chain A and B coupled together is a manifestation of the conventional CNHSE.

As the system size *N* increases further beyond $N_0$, real eigenenergies with $|E| < 2\sqrt{t_{\textrm{L,A}}t_{\textrm{L,B}}}$ re-emerge above the critical energy $N_2$. These real eigenenergies emerge because the $\Gamma |\beta _{\textrm{A}}|^N |\beta _{\textrm{B}}|^{2N} c_{1,1,2,0}$ term in the second row of Eq. (44) has now become large enough to cancel off the $|\beta _{\textrm{B}}|^{2N} t_{\textrm{C}}^2 c_{0,2,0,2}$ term in the first row of Eq. (44) to make $D_{|E| < 2\sqrt{t_{\textrm{L,A}}t_{\textrm{L,B}}}}$ zero. (The former has an extra factor of $|\beta _{\textrm{A}}|^N$ compared to the latter). $N_2$ is therefore given by

49 $$\begin{aligned} N_2 \simeq \frac{\textrm{Ln}\left( \frac{t_{\textrm{C}}^2}{\Gamma }\right) }{\textrm{Ln} |\beta _{\textrm{A}}|} \approx \frac{\textrm{Ln}\left( \frac{t_{\textrm{C}}^2}{\Gamma }\right) }{\textrm{Ln} \sqrt{\frac{t_{\textrm{L,A}}}{t_{\textrm{R,A}}}}}. \end{aligned}$$

The dependence of $N_3$ on $\Gamma$ shows that the reemergence of the real energy eigenvalues is a direct consequence of the terminal coupling. Finally, as *N* increases even further, the $\Gamma ^2 |\beta _{\textrm{A}}|^{2N}|\beta _{\textrm{B}}|^{2N}$ terms in the third line of Eq. (44) play an increasingly dominant role. The real eigenvalues disappear again when the $\Gamma ^2 |\beta _{\textrm{A}}|^{2N}|\beta _{\textrm{B}}|^{2N}$ become too large to be canceled off by the $\Gamma |\beta _{\textrm{A}}|^{N}|\beta _{\textrm{B}}|^{2N}$ terms (the former has an extra factor of $\Gamma |\beta _{\textrm{A}}|^N$) and $D_{|E| < 2\sqrt{t_{\textrm{L,A}}t_{\textrm{L,B}}}}$ can no longer be 0. This occurs at the critical system size of

50 $$\begin{aligned} N_3 \simeq -\frac{\textrm{Ln}\Gamma }{\textrm{Ln} |\beta _{\textrm{A}}|} \approx -\frac{\textrm{Ln}\Gamma }{\textrm{Ln} \sqrt{\frac{t_{\textrm{L,A}}}{t_{\textrm{R,A}}}}}. \end{aligned}$$

We remark briefly on the behavior of the real energy spectrum at $2\sqrt{t_{\textrm{L,A}}t_{\textrm{R,A}}}< E < 2\sqrt{t_{\textrm{L,B}}t_{\textrm{R,B}}}$. Within this energy range, the $\beta _{\textrm{B},\pm }$s still form a complex conjugate pair because the $E^2-4t_{\textrm{L,A}}t_{\textrm{L,B}}$ terms inside the square root in Eq. (41) are negative, whereas the $\beta _{\textrm{A},\pm }$s no longer form a complex conjugate pair because the terms inside the square root are now positive. Instead, for positive real *E*, the $\beta _{\textrm{A},\pm }$s are two distinct positive real numbers, which we label as $\beta _{\textrm{A},\pm }$. We analyze the behavior of the real eigenenergies at positive values slightly smaller than $E=2\sqrt{t_{\textrm{L,B}}t_{\textrm{R,B}}}$, at which $\beta _{\textrm{A},-}< 1< |\beta _{\textrm{B}}| < \beta _{\textrm{A},+}$ (Fig. 8b). Note that because the middle two of the four $|\beta |$ values have the same magnitude, the real energy line between $2\sqrt{t_{\textrm{L,A}}t_{\textrm{R,A}}}< |E| < 2\sqrt{t_{\textrm{L,B}}t_{\textrm{R,B}}}$ lies on the complex-energy plane GBZ of the system when the effects of $\Gamma$ are negligible.

In this case, the leading terms in the determinant are now

51 $$\begin{aligned} D_{E > 2\sqrt{t_{\textrm{L,A}}t_{\textrm{R,A}}}} =&\left[ \beta _{\textrm{A},+}^N |\beta _{\textrm{B}}|^N (c'_{0,1,0,1,0} + c'_{0,1,0,1,2}t_{\textrm{C}}^2 + c'_{0,1,0,1,4} t_{\textrm{C}}^4 ) + |\beta _{\textrm{B}}|^{2N} c'_{0,0,0,2,2} t_{\textrm{C}}^2 \right. \nonumber \\&+ \ \left. \beta _{\textrm{A},-}^N |\beta _{\textrm{B}}|^N (c'_{0,0,1,1,0} + c'_{0,0,1,1,2}t_{\textrm{C}}^2 + c'_{0,0,1,1,4} t_{\textrm{C}}^4 ) \right] \nonumber \\&+ \Gamma \left[ \beta _{\textrm{A},+}^N|\beta _{\textrm{B}}|^{2N} ( c'_{1,0,0,2,0} + c'_{1,0,0,2,2}t_{\textrm{C}}^2 + \cdots ) + |\beta _{\textrm{B}}|^{2N} ( c'_{1,0,0,2,0} + c'_{1,0,0,2,2}t_{\textrm{C}}^2 + \cdots ) + \cdots \right] \nonumber \\&+ \Gamma ^2 ( \ldots ) + \Gamma ^4 (\ldots ) \end{aligned}$$

where the $c'_{p_{\Gamma },p_{\beta _{\textrm{A},+}},p_{\beta _{\textrm{A},-}},p_{|\beta _{\textrm{B}}}, p_{t_{\textrm{C}}}}$ are the analogues of the $c_{p_\Gamma , p_{|\beta _{\textrm{A}}|}, p_{|\beta _{\textrm{B}}|}, p_{t_{\textrm{C}}}}$s in Eq. (44) except that there are now separate indices for $\beta _{\textrm{A},+}$ and $\beta _{\textrm{A},-}$. Note that because now $\beta _{\textrm{A},+}$ is now larger than $|\beta _{\textrm{B}}|$, the leading terms in Eq. (51) for each value of $p_{\Gamma }$ is not the one with the highest power of $|\beta _{\textrm{B}}|$, as was the case in Eq. (44), but rather the one with the highest power of $\beta _{\textrm{A},+}$. (The highest power of $\beta _{\textrm{A},+}$ in the omitted terms for $\Gamma ^3$ and $\Gamma ^4$ is $\beta _{\textrm{A},+}^N$.) Because the leading terms in $p_{\Gamma } = 0$ (i.e., the $\beta _{\textrm{A},+}^N|\beta _{\textrm{B}}|^N c'_{0,0,1,1,0}$ term in the first line) and $p_{\Gamma }=1$ (i.e., the $\beta _{\textrm{A},+}^N|\beta _{\textrm{B}}|^{2N} c'_{1,0,1,1,0}$ term on the third line) are both independent of $t_{\textrm{C}}$, we can neglect the contributions of $t_{\textrm{C}}$ and higher powers of $\Gamma$, and approximate $D_{2\sqrt{t_{\textrm{L,A}}t_{\textrm{R,A}}}}$ as

52 $$\begin{aligned} D_{E > 2\sqrt{t_{\textrm{L,A}}t_{\textrm{R,A}}}} \approx \beta _{\textrm{A},+}^N|\beta _{\textrm{B}}|^N ( c'_{0,1,0,1,0} + \Gamma |\beta _{\textrm{B}}|^N c'_{1,1,0,2,0}) \end{aligned}$$

When *N* becomes sufficiently large enough that $\Gamma |\beta _{\textrm{B}}|^N \gg 1$, the second term in Eq. (52) becomes too large to be canceled off by the first term for $D_{E > 2\sqrt{t_{\textrm{L,A}}t_{\textrm{R,A}}}}$ to become zero. The critical size $N_1$ beyond which real eigenenergies no longer exist at $2\sqrt{t_{\textrm{L,A}}t_{\textrm{R,A}}}< E < 2\sqrt{t_{\textrm{L,B}}t_{\textrm{R,B}}}$ is hence given by

53 $$\begin{aligned} N_1 \simeq -\frac{\textrm{Ln}\Gamma }{\textrm{Ln}|\beta _{\textrm{B}}|} \approx -\frac{\textrm{Ln}\Gamma }{\textrm{Ln}|\sqrt{t_{\textrm{L,B}}/t_{\textrm{R,B}}}}. \end{aligned}$$

### Full expression of $D_{|E| < 2\sqrt{t_{\textrm{L,A}}t_{\textrm{L,B}}}}$

The determinant $D_{|E| < 2\sqrt{t_{\textrm{L,A}}t_{\textrm{L,B}}}}$ is given by

54 $$\begin{aligned} D_{|E| < 2\sqrt{t_{\textrm{L,A}}t_{\textrm{L,B}}}} = g_0 + \Gamma g_1 + \Gamma ^2 g_2 + \Gamma ^3 g_3 + \Gamma ^4 g_4 \end{aligned}$$

where

55 $$\begin{aligned} g_0=&-|\beta _{\textrm{B}}|^{2 N+2}t_{\textrm{L,A}} t_{\textrm{L,B}} t_{\textrm{R,A}} t_{\textrm{R,B}}\left( \textrm{Re}\left( \chi _{\textrm{A},1} \chi _{\textrm{B},1}-\chi _{\textrm{A},2} \chi _{\textrm{B},1}\right) \right) \nonumber \\&+|\beta _{\textrm{A}}|^{N+1} |\beta _{\textrm{B}}|^{N+1}2 t_{\textrm{L,A}} t_{\textrm{L,B}} t_{\textrm{R,A}} t_{\textrm{R,B}}\Big (\textrm{Re}\big (\textrm{exp}(i (N+1) (k_{\textrm{A}}-k_{\textrm{B}}))-\textrm{exp}(i (N+1) (k_{\textrm{A}}+k_{\textrm{B}}))\nonumber \\&+\chi _{\textrm{A},1} \chi _{\textrm{B},1} \textrm{exp}(i (N+1) (k_{\textrm{A}}+k_{\textrm{B}}))+\chi _{\textrm{A},2} \chi _{\textrm{B},1} \left( -\textrm{exp}(i (N+1) (k_{\textrm{A}}-k_{\textrm{B}}))\right) +\chi _{\textrm{A},1} \chi _{\textrm{B},2} \left( -\textrm{exp}(i (N+1) (k_{\textrm{A}}-k_{\textrm{B}}))\right) \nonumber \\&+\chi _{\textrm{A},2} \chi _{\textrm{B},2} \textrm{exp}(i (N+1) (k_{\textrm{A}}+k_{\textrm{B}}))+\chi _{\textrm{A},1} \chi _{\textrm{A},2} \chi _{\textrm{B},1} \chi _{\textrm{B},2} \textrm{exp}(i (N+1) (k_{\textrm{A}}-k_{\textrm{B}}))\nonumber \\&+\chi _{\textrm{A},1} \chi _{\textrm{A},2} \chi _{\textrm{B},1} \chi _{\textrm{B},2} \left( -\textrm{exp}(i (N+1) (k_{\textrm{A}}+k_{\textrm{B}}))\right) \big )\Big )\nonumber \\&-|\beta _{\textrm{A}}|^{2 N+2}t_{\textrm{L,A}} t_{\textrm{L,B}} t_{\textrm{R,A}} t_{\textrm{R,B}}\Big (\textrm{Re}\big (\chi _{\textrm{A},1} \chi _{\textrm{B},1}-\chi _{\textrm{A},2} \chi _{\textrm{B},1}\big )\Big ) \end{aligned}$$

56 $$\begin{aligned} g_1=&|\beta _{\textrm{A}}|^{N} |\beta _{\textrm{B}}|^{2 N+1}2 t_{\textrm{R,A}} t_{\textrm{R,B}}\Big (|\beta _{\textrm{B}}| t_{\textrm{L,A}}\textrm{Re}\big (\chi _{\textrm{A},1} \chi _{\textrm{B},1} \textrm{exp}(i k_{\textrm{A}} N)+\chi _{\textrm{A},1} \chi _{\textrm{B},2} \left( -\textrm{exp}(i k_{\textrm{A}} N)\right) \big )\nonumber \\&+|\beta _{\textrm{A}}| t_{\textrm{L,A}}\textrm{Re}\big (-\textrm{exp}(i (k_{\textrm{A}} N+k_{\textrm{A}}-k_{\textrm{B}}))+\textrm{exp}(i (k_{\textrm{A}} N+k_{\textrm{A}}+k_{\textrm{B}}))+\chi _{\textrm{A},1} \chi _{\textrm{B},1} \left( -\textrm{exp}(i (k_{\textrm{A}} N+k_{\textrm{A}}+k_{\textrm{B}}))\right) \nonumber \\&+\chi _{\textrm{A},1} \chi _{\textrm{B},2} \textrm{exp}(i (k_{\textrm{A}} N+k_{\textrm{A}}-k_{\textrm{B}}))\big )+|\beta _{\textrm{B}}| t_{\textrm{L,B}}\textrm{Re}\big (\chi _{\textrm{A},2} \chi _{\textrm{B},1} \left( -\textrm{exp}(i k_{\textrm{A}} N)\right) +\chi _{\textrm{A},2} \chi _{\textrm{B},2} \textrm{exp}(i k_{\textrm{A}} N)\big )\nonumber \\&+|\beta _{\textrm{A}}| t_{\textrm{L,B}}\textrm{Re}\big (\chi _{\textrm{A},2} \chi _{\textrm{B},1} \textrm{exp}(i (k_{\textrm{A}} N+k_{\textrm{A}}-k_{\textrm{B}}))+\chi _{\textrm{A},2} \chi _{\textrm{B},2} \left( -\textrm{exp}(i (k_{\textrm{A}} N+k_{\textrm{A}}+k_{\textrm{B}}))\right) \nonumber \\&+\chi _{\textrm{A},1} \chi _{\textrm{A},2} \chi _{\textrm{B},1} \chi _{\textrm{B},2} \left( -\textrm{exp}(i (k_{\textrm{A}} N+k_{\textrm{A}}-k_{\textrm{B}}))\right) +\chi _{\textrm{A},1} \chi _{\textrm{A},2} \chi _{\textrm{B},1} \chi _{\textrm{B},2} \textrm{exp}(i (k_{\textrm{A}} N+k_{\textrm{A}}+k_{\textrm{B}}))\big )\Big )\nonumber \\&+|\beta _{\textrm{A}}|^{2 N+1} |\beta _{\textrm{B}}|^{N}2 t_{\textrm{R,A}} t_{\textrm{R,B}}\Big (|\beta _{\textrm{A}}| t_{\textrm{L,B}}\textrm{Re}\big (\chi _{\textrm{A},1} \chi _{\textrm{B},1} \textrm{exp}(i k_{\textrm{B}} N)+\chi _{\textrm{A},2} \chi _{\textrm{B},1} \left( -\textrm{exp}(i k_{\textrm{B}} N)\right) \big )\nonumber \\&+|\beta _{\textrm{B}}| t_{\textrm{L,B}}\textrm{Re}\big (\textrm{exp}(i (k_{\textrm{A}}+k_{\textrm{B}} N+k_{\textrm{B}}))-\textrm{exp}(i (k_{\textrm{A}}-k_{\textrm{B}} (N+1)))+\chi _{\textrm{A},1} \chi _{\textrm{B},1} \left( -\textrm{exp}(i (k_{\textrm{A}}+k_{\textrm{B}} N+k_{\textrm{B}}))\right) \nonumber \\&+\chi _{\textrm{A},1} \chi _{\textrm{B},2} \textrm{exp}(i (k_{\textrm{A}}-k_{\textrm{B}} (N+1)))\big )+|\beta _{\textrm{A}}| t_{\textrm{L,A}}\textrm{Re}\big (\chi _{\textrm{A},1} \chi _{\textrm{B},2} \left( -\textrm{exp}(i k_{\textrm{B}} N)\right) +\chi _{\textrm{A},2} \chi _{\textrm{B},2} \textrm{exp}(i k_{\textrm{B}} N)\big )\nonumber \\&+|\beta _{\textrm{B}}| t_{\textrm{L,A}}\textrm{Re}\big (\chi _{\textrm{A},2} \chi _{\textrm{B},1} \textrm{exp}(i (k_{\textrm{A}}-k_{\textrm{B}} (N+1)))+\chi _{\textrm{A},2} \chi _{\textrm{B},2} \left( -\textrm{exp}(i (k_{\textrm{A}}+k_{\textrm{B}} N+k_{\textrm{B}}))\right) \nonumber \\&+\chi _{\textrm{A},1} \chi _{\textrm{A},2} \chi _{\textrm{B},1} \chi _{\textrm{B},2} \textrm{exp}(i (k_{\textrm{A}}+k_{\textrm{B}} N+k_{\textrm{B}}))+\chi _{\textrm{A},1} \chi _{\textrm{A},2} \chi _{\textrm{B},1} \chi _{\textrm{B},2} \left( -\textrm{exp}(i (k_{\textrm{A}}-k_{\textrm{B}} (N+1)))\right) \big )\Big )\nonumber \\&+|\beta _{\textrm{B}}|^{N+1}2 t_{\textrm{L,A}} t_{\textrm{L,B}}\Big (|\beta _{\textrm{B}}| t_{\textrm{R,A}}\textrm{Re}\big (\chi _{\textrm{A},1} \chi _{\textrm{B},1} \textrm{exp}(i k_{\textrm{B}} N)+\chi _{\textrm{A},2} \chi _{\textrm{B},1} \left( -\textrm{exp}(i k_{\textrm{B}} N)\right) \big )\nonumber \\&+|\beta _{\textrm{B}}| t_{\textrm{R,B}}\textrm{Re}\big (\chi _{\textrm{A},1} \chi _{\textrm{B},2} \left( -\textrm{exp}(i k_{\textrm{B}} N)\right) +\chi _{\textrm{A},2} \chi _{\textrm{B},2} \textrm{exp}(i k_{\textrm{B}} N)\big )+|\beta _{\textrm{A}}| t_{\textrm{R,B}}\textrm{Re}\big (\nonumber \\&\textrm{exp}(i (k_{\textrm{A}}+k_{\textrm{B}} N+k_{\textrm{B}}))-\textrm{exp}(i (k_{\textrm{A}}-k_{\textrm{B}} (N+1)))+\chi _{\textrm{A},2} \chi _{\textrm{B},1} \textrm{exp}(i (k_{\textrm{A}}-k_{\textrm{B}} (N+1)))\nonumber \\&+\chi _{\textrm{A},2} \chi _{\textrm{B},2} \left( -\textrm{exp}(i (k_{\textrm{A}}+k_{\textrm{B}} N+k_{\textrm{B}}))\right) \big )+|\beta _{\textrm{A}}| t_{\textrm{R,A}}\textrm{Re}\big (\chi _{\textrm{A},1} \chi _{\textrm{B},1} \left( -\textrm{exp}(i (k_{\textrm{A}}+k_{\textrm{B}} N+k_{\textrm{B}}))\right) \nonumber \\&+\chi _{\textrm{A},1} \chi _{\textrm{B},2} \textrm{exp}(i (k_{\textrm{A}}-k_{\textrm{B}} (N+1)))+\chi _{\textrm{A},1} \chi _{\textrm{A},2} \chi _{\textrm{B},1} \chi _{\textrm{B},2} \textrm{exp}(i (k_{\textrm{A}}+k_{\textrm{B}} N+k_{\textrm{B}}))\nonumber \\&+\chi _{\textrm{A},1} \chi _{\textrm{A},2} \chi _{\textrm{B},1} \chi _{\textrm{B},2} \left( -\textrm{exp}(i (k_{\textrm{A}}-k_{\textrm{B}} (N+1)))\right) \big )\Big )\nonumber \\&+|\beta _{\textrm{A}}|^{N+1}-2 t_{\textrm{L,A}} t_{\textrm{L,B}}\Big (|\beta _{\textrm{A}}| t_{\textrm{R,B}}\textrm{Re}\big (\chi _{\textrm{A},1} \chi _{\textrm{B},1} \left( -\textrm{exp}(i k_{\textrm{A}} N)\right) +\chi _{\textrm{A},1} \chi _{\textrm{B},2} \textrm{exp}(i k_{\textrm{A}} N)\big )\nonumber \\&+|\beta _{\textrm{A}}| t_{\textrm{R,A}}\textrm{Re}\big (\chi _{\textrm{A},2} \chi _{\textrm{B},1} \textrm{exp}(i k_{\textrm{A}} N)+\chi _{\textrm{A},2} \chi _{\textrm{B},2} \left( -\textrm{exp}(i k_{\textrm{A}} N)\right) \big )+|\beta _{\textrm{B}}| t_{\textrm{R,A}}\textrm{Re}\big (\nonumber \\&-\textrm{exp}(i (k_{\textrm{A}} N+k_{\textrm{A}}+k_{\textrm{B}}))+\chi _{\textrm{A},2} \chi _{\textrm{B},2} \textrm{exp}(i (k_{\textrm{A}} N+k_{\textrm{A}}+k_{\textrm{B}}))\big )+|\beta _{\textrm{B}}| t_{\textrm{R,B}}\textrm{Re}\big (\chi _{\textrm{A},1} \chi _{\textrm{B},1} \textrm{exp}(i (k_{\textrm{A}} N+k_{\textrm{A}}+k_{\textrm{B}}))\nonumber \\&+\chi _{\textrm{A},1} \chi _{\textrm{A},2} \chi _{\textrm{B},1} \chi _{\textrm{B},2} \left( -\textrm{exp}(i (k_{\textrm{A}} N+k_{\textrm{A}}+k_{\textrm{B}}))\right) \big )\Big ) \end{aligned}$$

57 $$\begin{aligned} g_2=&-|\beta _{\textrm{A}}|^{2 N} |\beta _{\textrm{B}}|^{2 N}t_{\textrm{R,A}} t_{\textrm{R,B}}\Big (|\beta _{\textrm{A}}|^2\textrm{Re}\big (\chi _{\textrm{A},1} \chi _{\textrm{B},1}-\chi _{\textrm{A},2} \chi _{\textrm{B},1}\big )+|\beta _{\textrm{B}}|^2\textrm{Re}\big (\chi _{\textrm{A},1} \chi _{\textrm{B},1}\nonumber \\&-\chi _{\textrm{A},2} \chi _{\textrm{B},1}\big )-2 |\beta _{\textrm{A}}| |\beta _{\textrm{B}}|\textrm{Re}\big (\textrm{exp}(i (k_{\textrm{A}}-k_{\textrm{B}}))+\chi _{\textrm{A},1} \chi _{\textrm{B},1} \textrm{exp}(i (k_{\textrm{A}}+k_{\textrm{B}}))\nonumber \\&+\chi _{\textrm{A},2} \chi _{\textrm{B},2} \textrm{exp}(i (k_{\textrm{A}}+k_{\textrm{B}}))+\chi _{\textrm{A},1} \chi _{\textrm{A},2} \chi _{\textrm{B},1} \chi _{\textrm{B},2} \textrm{exp}(i (k_{\textrm{A}}-k_{\textrm{B}}))\big )+2 |\beta _{\textrm{A}}| |\beta _{\textrm{B}}|\textrm{Re}\big (\nonumber \\&\textrm{exp}(i (k_{\textrm{A}}+k_{\textrm{B}}))+\chi _{\textrm{A},2} \chi _{\textrm{B},1} \textrm{exp}(i (k_{\textrm{A}}-k_{\textrm{B}}))+\chi _{\textrm{A},1} \chi _{\textrm{B},2} \textrm{exp}(i (k_{\textrm{A}}-k_{\textrm{B}}))\nonumber \\&+\chi _{\textrm{A},1} \chi _{\textrm{A},2} \chi _{\textrm{B},1} \chi _{\textrm{B},2} \textrm{exp}(i (k_{\textrm{A}}+k_{\textrm{B}}))\big )\Big )\nonumber \\&+|\beta _{\textrm{A}}| |\beta _{\textrm{B}}|^{2 N+1}2\Big (t_{\textrm{L,A}} t_{\textrm{R,B}}\textrm{Re}\big (\textrm{exp}(i (k_{\textrm{A}}-k_{\textrm{B}}))-\textrm{exp}(i (k_{\textrm{A}}+k_{\textrm{B}}))\big )+t_{\textrm{L,A}} t_{\textrm{R,A}}\textrm{Re}\big (\nonumber \\&\chi _{\textrm{A},1} \chi _{\textrm{B},1} \textrm{exp}(i (k_{\textrm{A}}+k_{\textrm{B}}))+\chi _{\textrm{A},1} \chi _{\textrm{B},2} \left( -\textrm{exp}(i (k_{\textrm{A}}-k_{\textrm{B}}))\right) \big )+t_{\textrm{L,B}} t_{\textrm{R,B}}\textrm{Re}\big (\nonumber \\&\chi _{\textrm{A},2} \chi 
_{\textrm{B},1} \left( -\textrm{exp}(i (k_{\textrm{A}}-k_{\textrm{B}}))\right) +\chi _{\textrm{A},2} \chi _{\textrm{B},2} \textrm{exp}(i (k_{\textrm{A}}+k_{\textrm{B}}))\big )+t_{\textrm{L,B}} t_{\textrm{R,A}}\textrm{Re}\big (\nonumber \\&\chi _{\textrm{A},1} \chi _{\textrm{A},2} \chi _{\textrm{B},1} \chi _{\textrm{B},2} \textrm{exp}(i (k_{\textrm{A}}-k_{\textrm{B}}))+\chi _{\textrm{A},1} \chi _{\textrm{A},2} \chi _{\textrm{B},1} \chi _{\textrm{B},2} \left( -\textrm{exp}(i (k_{\textrm{A}}+k_{\textrm{B}}))\right) \big )\Big )\nonumber \\&+|\beta _{\textrm{A}}|^{N} |\beta _{\textrm{B}}|^{N}2\Big (-|\beta _{\textrm{B}}|^2 t_{\textrm{L,A}} t_{\textrm{R,A}}\textrm{Re}\big (\textrm{exp}(i N (k_{\textrm{A}}+k_{\textrm{B}}))+\chi _{\textrm{A},1}+\chi _{\textrm{B},1}\big )+|\beta _{\textrm{B}}|^2 t_{\textrm{L,B}} t_{\textrm{R,A}}\textrm{Re}\big (\textrm{exp}(i N (k_{\textrm{A}}+k_{\textrm{B}}))+ \nonumber \\&\chi _{\textrm{A},2}+\chi _{\textrm{B},1}\big ) +|\beta _{\textrm{B}}|^2 t_{\textrm{L,A}} t_{\textrm{R,B}}\textrm{Re}\big (\textrm{exp}(i N (k_{\textrm{A}}+k_{\textrm{B}}))+\chi _{\textrm{A},1}+\chi _{\textrm{B},2}\big )\nonumber \\&-|\beta _{\textrm{B}}|^2 t_{\textrm{L,B}} t_{\textrm{R,B}}\textrm{Re}\big (\textrm{exp}(i N (k_{\textrm{A}}+k_{\textrm{B}}))+\chi _{\textrm{A},2}+\chi _{\textrm{B},2}\big )+|\beta _{\textrm{A}}|^2 t_{\textrm{L,B}} t_{\textrm{R,B}}\textrm{Re}\big (\nonumber \\&\chi _{\textrm{A},1} \chi _{\textrm{B},1} \left( -\textrm{exp}(i N (k_{\textrm{A}}+k_{\textrm{B}}))\right) +\chi _{\textrm{A},1} \chi _{\textrm{B},2} \textrm{exp}(i N (k_{\textrm{A}}-k_{\textrm{B}}))\big )+|\beta _{\textrm{A}}|^2 t_{\textrm{L,A}} t_{\textrm{R,B}}\textrm{Re}\big (\nonumber \\&\chi _{\textrm{A},1} \chi _{\textrm{B},1} \left( -\textrm{exp}(i N (k_{\textrm{A}}-k_{\textrm{B}}))\right) +\chi _{\textrm{A},1} \chi _{\textrm{B},2} \textrm{exp}(i N (k_{\textrm{A}}+k_{\textrm{B}}))\big )+|\beta _{\textrm{A}}| |\beta _{\textrm{B}}| t_{\textrm{L,A}} t_{\textrm{R,B}}\textrm{Re}\big (\nonumber \\&\chi _{\textrm{A},1} \chi _{\textrm{B},1} \textrm{exp}(i (k_{\textrm{A}} N+k_{\textrm{A}}-k_{\textrm{B}} N+k_{\textrm{B}}))+\chi _{\textrm{A},1} \chi _{\textrm{B},2} \left( -\textrm{exp}(i (k_{\textrm{A}} N+k_{\textrm{A}}+k_{\textrm{B}} N-k_{\textrm{B}}))\right) \nonumber \\&+\chi _{\textrm{A},1} \chi _{\textrm{B},2} \left( -\textrm{exp}(i (k_{\textrm{A}} N-k_{\textrm{A}}+k_{\textrm{B}} N+k_{\textrm{B}}))\right) \big )\nonumber \\&+|\beta _{\textrm{A}}|^2 t_{\textrm{L,B}} t_{\textrm{R,A}}\textrm{Re}\big (\chi _{\textrm{A},2} \chi _{\textrm{B},1} \textrm{exp}(i N (k_{\textrm{A}}+k_{\textrm{B}}))+\chi _{\textrm{A},2} \chi _{\textrm{B},2} \left( -\textrm{exp}(i N (k_{\textrm{A}}-k_{\textrm{B}}))\right) \big ) \nonumber \\&+|\beta _{\textrm{A}}|^2 t_{\textrm{L,A}} t_{\textrm{R,A}}\textrm{Re} \chi _{\textrm{A},2} \chi _{\textrm{B},1} \textrm{exp}(i N (k_{\textrm{A}}-k_{\textrm{B}}))+\chi _{\textrm{A},2} \chi _{\textrm{B},2} \left( -\textrm{exp}(i N (k_{\textrm{A}}+k_{\textrm{B}}))\right) \big )+|\beta _{\textrm{A}}| |\beta _{\textrm{B}}| t_{\textrm{L,B}} t_{\textrm{R,A}}\textrm{Re}\big (\nonumber \\&\chi _{\textrm{A},2} \chi _{\textrm{B},1} \left( -\textrm{exp}(i (k_{\textrm{A}} N+k_{\textrm{A}}+k_{\textrm{B}} N-k_{\textrm{B}}))\right) \nonumber \\&+\chi _{\textrm{A},2} \chi _{\textrm{B},1} \left( -\textrm{exp}(i (k_{\textrm{A}} N-k_{\textrm{A}}+k_{\textrm{B}} N+k_{\textrm{B}}))\right) +\chi _{\textrm{A},2} \chi _{\textrm{B},2} \textrm{exp}(i (k_{\textrm{A}} N+k_{\textrm{A}}-k_{\textrm{B}} N+k_{\textrm{B}}))\big )\nonumber \\&+|\beta _{\textrm{A}}| |\beta _{\textrm{B}}| t_{\textrm{L,B}} t_{\textrm{R,B}}\textrm{Re}\big (\textrm{exp}(i (k_{\textrm{A}} N-k_{\textrm{A}}+k_{\textrm{B}} N+k_{\textrm{B}}))+\chi _{\textrm{A},1} \chi _{\textrm{A},2} \chi _{\textrm{B},1} \chi _{\textrm{B},2} \left( -\textrm{exp}(i (k_{\textrm{A}} N+k_{\textrm{A}}-k_{\textrm{B}} N+k_{\textrm{B}}))\right) \nonumber \\&+\chi _{\textrm{A},1} \chi _{\textrm{A},2} \chi _{\textrm{B},1} \chi _{\textrm{B},2} \textrm{exp}(i (k_{\textrm{A}} N+k_{\textrm{A}}+k_{\textrm{B}} N-k_{\textrm{B}}))\big )+|\beta _{\textrm{A}}| |\beta _{\textrm{B}}| t_{\textrm{L,A}} t_{\textrm{R,A}}\textrm{Re}\big (-\textrm{exp}(i (k_{\textrm{A}} N+k_{\textrm{A}}-k_{\textrm{B}} N+k_{\textrm{B}}))\nonumber \\&+\textrm{exp}(i (k_{\textrm{A}} N+k_{\textrm{A}}+k_{\textrm{B}} N-k_{\textrm{B}}))+\chi _{\textrm{A},1} \chi _{\textrm{A},2} \chi _{\textrm{B},1} \chi _{\textrm{B},2} \textrm{exp}(i (k_{\textrm{A}} N-k_{\textrm{A}}+k_{\textrm{B}} N+k_{\textrm{B}}))\big )\Big )\nonumber \\&|\beta _{\textrm{B}}| \left( -|\beta _{\textrm{A}}|^{2 N+1}\right) 2\Big (t_{\textrm{L,B}} t_{\textrm{R,A}}\textrm{Re}\big (e+i (k_{\textrm{A}}+k_{\textrm{B}})\big )-t_{\textrm{L,B}} t_{\textrm{R,B}}\textrm{Re}\big (\textrm{exp}(i (k_{\textrm{A}}+k_{\textrm{B}}))+\chi _{\textrm{A},1}+\chi _{\textrm{B},1}\big )-t_{\textrm{L,A}} t_{\textrm{R,A}}\textrm{Re}\big (\nonumber \\&\textrm{exp}(i (k_{\textrm{A}}+k_{\textrm{B}}))+\chi _{\textrm{A},2}+\chi _{\textrm{B},2}\big )+t_{\textrm{L,A}} t_{\textrm{R,B}}\textrm{Re}\big (\textrm{exp}(i (k_{\textrm{A}}+k_{\textrm{B}}))+\chi _{\textrm{A},1}+\chi _{\textrm{A},2}+\chi _{\textrm{B},1}+\chi _{\textrm{B},2}\big )\Big )\nonumber \\&+t_{\textrm{L,A}} t_{\textrm{L,B}}\Big (-|\beta _{\textrm{A}}|^2-|\beta _{\textrm{B}}|^2\textrm{Re}\big (\chi _{\textrm{A},1}+\chi _{\textrm{B},1}\big )+|\beta _{\textrm{A}}|^2\textrm{Re}\big (\chi _{\textrm{A},2}+\chi _{\textrm{B},1}\big )+|\beta _{\textrm{B}}|^2\textrm{Re}\big (\chi _{\textrm{A},2}+\chi _{\textrm{B},1}\big )+\nonumber \\&2 |\beta _{\textrm{A}}| |\beta _{\textrm{B}}|\textrm{Re}\big (\textrm{exp}(i (k_{\textrm{A}}-k_{\textrm{B}})) +\chi _{\textrm{A},1} \chi _{\textrm{B},1} \textrm{exp}(i (k_{\textrm{A}}+k_{\textrm{B}}))+\chi _{\textrm{A},2} \chi _{\textrm{B},2} \textrm{exp}(i (k_{\textrm{A}}+k_{\textrm{B}}))+\chi _{\textrm{A},1} \chi _{\textrm{A},2} \chi _{\textrm{B},1} \chi _{\textrm{B},2} \textrm{exp}(i (k_{\textrm{A}}-k_{\textrm{B}}))\big )\nonumber \\&-2 |\beta _{\textrm{A}}| |\beta _{\textrm{B}}|\textrm{Re}\big (\textrm{exp}(i (k_{\textrm{A}}+k_{\textrm{B}}))+\chi _{\textrm{A},2} \chi _{\textrm{B},1} \textrm{exp}(i (k_{\textrm{A}}-k_{\textrm{B}}))+\chi _{\textrm{A},1} \chi _{\textrm{B},2} \textrm{exp}(i (k_{\textrm{A}}-k_{\textrm{B}}))\nonumber \\&+\chi _{\textrm{A},1} \chi _{\textrm{A},2} \chi _{\textrm{B},1} \chi _{\textrm{B},2} \textrm{exp}(i (k_{\textrm{A}}+k_{\textrm{B}}))\big )\Big ) \end{aligned}$$

58 $$\begin{aligned} g_3=&|\beta _{\textrm{A}}|^{N+1} |\beta _{\textrm{B}}|^{2 N}2\Big (|\beta _{\textrm{A}}| t_{\textrm{R,B}}\textrm{Re}\big (\chi _{\textrm{A},1} \chi _{\textrm{B},1} \textrm{exp}(i k_{\textrm{A}} N)+\chi _{\textrm{A},1} \chi _{\textrm{B},2} \left( -\textrm{exp}(i k_{\textrm{A}} N)\right) \big )+|\beta _{\textrm{A}}| t_{\textrm{R,A}}\textrm{Re}\big (\nonumber \\&\chi _{\textrm{A},2} \chi _{\textrm{B},1} \left( -\textrm{exp}(i k_{\textrm{A}} N)\right) +\chi _{\textrm{A},2} \chi _{\textrm{B},2} \textrm{exp}(i k_{\textrm{A}} N)\big )+|\beta _{\textrm{B}}| t_{\textrm{R,B}}\textrm{Re}\big (-\textrm{exp}(i (k_{\textrm{A}} (N-1)+k_{\textrm{B}}))\nonumber \\&+\textrm{exp}(i (k_{\textrm{A}} (-N)+k_{\textrm{A}}+k_{\textrm{B}}))+\chi _{\textrm{A},1} \chi _{\textrm{B},2} \textrm{exp}(i (k_{\textrm{A}} (N-1)+k_{\textrm{B}}))+\chi _{\textrm{A},2} \chi _{\textrm{B},2} \left( -\textrm{exp}(i (k_{\textrm{A}} (-N)+k_{\textrm{A}}+k_{\textrm{B}}))\right) \big )\big (\nonumber \\&+|\beta _{\textrm{B}}| t_{\textrm{R,A}}\textrm{Re} \chi _{\textrm{A},1} \chi _{\textrm{B},1} \left( -\textrm{exp}(i (k_{\textrm{A}} (-N)+k_{\textrm{A}}+k_{\textrm{B}}))\right) +\chi _{\textrm{A},2} \chi _{\textrm{B},1} \textrm{exp}(i (k_{\textrm{A}} (N-1)+k_{\textrm{B}}))\nonumber \\&+\chi _{\textrm{A},1} \chi _{\textrm{A},2} \chi _{\textrm{B},1} \chi _{\textrm{B},2} \left( -\textrm{exp}(i (k_{\textrm{A}} (N-1)+k_{\textrm{B}}))\right) +\chi _{\textrm{A},1} \chi _{\textrm{A},2} \chi _{\textrm{B},1} \chi _{\textrm{B},2} \textrm{exp}(i (k_{\textrm{A}} (-N)+k_{\textrm{A}}+k_{\textrm{B}}))\big )\Big )\nonumber \\&+|\beta _{\textrm{A}}|^{2 N} |\beta _{\textrm{B}}|^{N+1}2\Big (|\beta _{\textrm{B}}| t_{\textrm{R,A}}\textrm{Re}\big (\chi _{\textrm{A},1} \chi _{\textrm{B},1} \textrm{exp}(i k_{\textrm{B}} N)+\chi _{\textrm{A},2} \chi _{\textrm{B},1} \left( -\textrm{exp}(i k_{\textrm{B}} N)\right) \big )+|\beta _{\textrm{B}}| t_{\textrm{R,B}}\textrm{Re}\big (\nonumber \\&\chi _{\textrm{A},1} \chi _{\textrm{B},2} \left( -\textrm{exp}(i k_{\textrm{B}} N)\right) +\chi _{\textrm{A},2} \chi _{\textrm{B},2} \textrm{exp}(i k_{\textrm{B}} N)\big )+|\beta _{\textrm{A}}| t_{\textrm{R,A}}\textrm{Re}\big (-\textrm{exp}(i (k_{\textrm{A}}+k_{\textrm{B}} (N-1)))\nonumber \\&+\textrm{exp}(i (k_{\textrm{A}}-k_{\textrm{B}} N+k_{\textrm{B}}))+\chi _{\textrm{A},2} \chi _{\textrm{B},1} \textrm{exp}(i (k_{\textrm{A}}+k_{\textrm{B}} (N-1)))+\chi _{\textrm{A},2} \chi _{\textrm{B},2} \left( -\textrm{exp}(i (k_{\textrm{A}}-k_{\textrm{B}} N+k_{\textrm{B}}))\right) \big )+|\beta _{\textrm{A}}| t_{\textrm{R,B}}\textrm{Re}\big (\nonumber \\&\chi _{\textrm{A},1} \chi _{\textrm{B},1} \left( -\textrm{exp}(i (k_{\textrm{A}}-k_{\textrm{B}} N+k_{\textrm{B}}))\right) +\chi _{\textrm{A},1} \chi _{\textrm{B},2} \textrm{exp}(i (k_{\textrm{A}}+k_{\textrm{B}} (N-1)))\nonumber \\&+\chi _{\textrm{A},1} \chi _{\textrm{A},2} \chi _{\textrm{B},1} \chi _{\textrm{B},2} \left( -\textrm{exp}(i (k_{\textrm{A}}+k_{\textrm{B}} (N-1)))\right) +\chi _{\textrm{A},1} \chi _{\textrm{A},2} \chi _{\textrm{B},1} \chi _{\textrm{B},2} \textrm{exp}(i (k_{\textrm{A}}-k_{\textrm{B}} N+k_{\textrm{B}}))\big )\Big )\nonumber \\&+|\beta _{\textrm{A}}| |\beta _{\textrm{B}}|^{N}2\Big (|\beta _{\textrm{A}}| t_{\textrm{L,B}}\textrm{Re}\big (\chi _{\textrm{A},1} \chi _{\textrm{B},1} \textrm{exp}(i k_{\textrm{B}} N)+\chi _{\textrm{A},2} \chi _{\textrm{B},1} \left( -\textrm{exp}(i k_{\textrm{B}} N)\right) \big )+|\beta _{\textrm{B}}| t_{\textrm{L,A}}\textrm{Re}\big (\nonumber \\&-\textrm{exp}(i (k_{\textrm{A}}+k_{\textrm{B}} (N-1)))+\textrm{exp}(i 
(k_{\textrm{A}}-k_{\textrm{B}} N+k_{\textrm{B}}))+\chi _{\textrm{A},1} \chi _{\textrm{B},1} \left( -\textrm{exp}(i (k_{\textrm{A}}-k_{\textrm{B}} N+k_{\textrm{B}}))\right) \nonumber \\&+\chi _{\textrm{A},1} \chi _{\textrm{B},2} \textrm{exp}(i (k_{\textrm{A}}+k_{\textrm{B}} (N-1)))\big )+|\beta _{\textrm{A}}| t_{\textrm{L,A}}\textrm{Re}\big (\chi _{\textrm{A},1} \chi _{\textrm{B},2} \left( -\textrm{exp}(i k_{\textrm{B}} N)\right) +\chi _{\textrm{A},2} \chi _{\textrm{B},2} \textrm{exp}(i k_{\textrm{B}} N)\big )\nonumber \\&+|\beta _{\textrm{B}}| t_{\textrm{L,B}}\textrm{Re}\big (\chi _{\textrm{A},2} \chi _{\textrm{B},1} \textrm{exp}(i (k_{\textrm{A}}+k_{\textrm{B}} (N-1)))+\chi _{\textrm{A},2} \chi _{\textrm{B},2} \left( -\textrm{exp}(i (k_{\textrm{A}}-k_{\textrm{B}} N+k_{\textrm{B}}))\right) \nonumber \\&+\chi _{\textrm{A},1} \chi _{\textrm{A},2} \chi _{\textrm{B},1} \chi _{\textrm{B},2} \left( -\textrm{exp}(i (k_{\textrm{A}}+k_{\textrm{B}} (N-1)))\right) +\chi _{\textrm{A},1} \chi _{\textrm{A},2} \chi _{\textrm{B},1} \chi _{\textrm{B},2} \textrm{exp}(i (k_{\textrm{A}}-k_{\textrm{B}} N+k_{\textrm{B}}))\big )\Big )\nonumber \\&|\beta _{\textrm{B}}| \left( -|\beta _{\textrm{A}}|^{N}\right) 2\Big (|\beta _{\textrm{A}}| t_{\textrm{L,B}}\textrm{Re}\big (\textrm{exp}(i (k_{\textrm{A}} (N-1)+k_{\textrm{B}}))-\textrm{exp}(i (k_{\textrm{A}} (-N)+k_{\textrm{A}}+k_{\textrm{B}}))+\chi _{\textrm{A},1} \chi _{\textrm{B},1} \textrm{exp}(i (k_{\textrm{A}} (-N)+k_{\textrm{A}}+k_{\textrm{B}}))\nonumber \\&+\chi _{\textrm{A},2} \chi _{\textrm{B},1} \left( -\textrm{exp}(i (k_{\textrm{A}} (N-1)+k_{\textrm{B}}))\right) \big )+|\beta _{\textrm{B}}| t_{\textrm{L,A}}\textrm{Re}\big (\chi _{\textrm{A},1} \chi _{\textrm{B},1} \left( -\textrm{exp}(i k_{\textrm{A}} N)\right) \nonumber \\&+\chi _{\textrm{A},1} \chi _{\textrm{B},2} \textrm{exp}(i k_{\textrm{A}} N)\big )+|\beta _{\textrm{B}}| t_{\textrm{L,B}}\textrm{Re}\big (\chi _{\textrm{A},2} \chi _{\textrm{B},1} \textrm{exp}(i k_{\textrm{A}} N)+\chi _{\textrm{A},2} \chi _{\textrm{B},2} \left( -\textrm{exp}(i k_{\textrm{A}} N)\right) \big )\nonumber \\&+|\beta _{\textrm{A}}| t_{\textrm{L,A}}\textrm{Re}\big (\chi _{\textrm{A},1} \chi _{\textrm{B},2} \left( -\textrm{exp}(i (k_{\textrm{A}} (N-1)+k_{\textrm{B}}))\right) +\chi _{\textrm{A},2} \chi _{\textrm{B},2} \textrm{exp}(i (k_{\textrm{A}} (-N)+k_{\textrm{A}}+k_{\textrm{B}}))\nonumber \\&+\chi _{\textrm{A},1} \chi _{\textrm{A},2} \chi _{\textrm{B},1} \chi _{\textrm{B},2} \textrm{exp}(i (k_{\textrm{A}} (N-1)+k_{\textrm{B}}))+\chi _{\textrm{A},1} \chi _{\textrm{A},2} \chi _{\textrm{B},1} \chi _{\textrm{B},2} \left( -\textrm{exp}(i (k_{\textrm{A}} (-N)+k_{\textrm{A}}+k_{\textrm{B}}))\right) \big )\Big ) \end{aligned}$$

59 $$\begin{aligned} g_4=&-|\beta _{\textrm{A}}|^2 |\beta _{\textrm{B}}|^{2 N}1\Big (\textrm{Re}\big (\chi _{\textrm{A},1} \chi _{\textrm{B},1}-\chi _{\textrm{A},2} \chi _{\textrm{B},1}\big )\Big )\nonumber \\&+|\beta _{\textrm{A}}|^{N+1} |\beta _{\textrm{B}}|^{N+1}2\Big (\textrm{Re}\big (\textrm{exp}(i (N-1) (k_{\textrm{A}}-k_{\textrm{B}}))-\textrm{exp}(i (N-1) (k_{\textrm{A}}+k_{\textrm{B}}))+\chi _{\textrm{A},1} \chi _{\textrm{B},1} \textrm{exp}(i (N-1) (k_{\textrm{A}}+k_{\textrm{B}}))\nonumber \\&+\chi _{\textrm{A},2} \chi _{\textrm{B},1} \left( -\textrm{exp}(i (N-1) (k_{\textrm{A}}-k_{\textrm{B}}))\right) +\chi _{\textrm{A},1} \chi _{\textrm{B},2} \left( -\textrm{exp}(i (N-1) (k_{\textrm{A}}-k_{\textrm{B}}))\right) +\chi _{\textrm{A},2} \chi _{\textrm{B},2} \textrm{exp}(i (N-1) (k_{\textrm{A}}+k_{\textrm{B}}))\nonumber \\&+\chi _{\textrm{A},1} \chi _{\textrm{A},2} \chi _{\textrm{B},1} \chi _{\textrm{B},2} \textrm{exp}(i (N-1) (k_{\textrm{A}}-k_{\textrm{B}}))+\chi _{\textrm{A},1} \chi _{\textrm{A},2} \chi _{\textrm{B},1} \chi _{\textrm{B},2} \left( -\textrm{exp}(i (N-1) (k_{\textrm{A}}+k_{\textrm{B}}))\right) \big )\Big )\nonumber \\&+|\beta _{\textrm{B}}|^2 \left( -|\beta _{\textrm{A}}|^{2 N}\right) 1\Big (\textrm{Re}\big (\chi _{\textrm{A},1} \chi _{\textrm{B},1}-\chi _{\textrm{A},2} \chi _{\textrm{B},1}\big )\Big ) \end{aligned}$$

## Data Availability

The data that support the plots and other findings of this study are available from the corresponding author upon reasonable request.

## Code availability

The computer codes used in the current study are accessible from the corresponding author upon reasonable request.
